# Characteristics of Fatty Acid Metabolism in Lung Adenocarcinoma to Guide Clinical Treatment

**DOI:** 10.3389/fimmu.2022.916284

**Published:** 2022-07-01

**Authors:** Dejing Huang, Enyu Tang, Tianze Zhang, Guangquan Xu

**Affiliations:** ^1^ The Second Affiliated Hospital of Harbin Medical University, Harbin, China; ^2^ Harbin Medical University, Harbin, China

**Keywords:** fatty acid metabolism, adenocarcinoma of lung cancer, tumor microenvironment, immunotherapy, chemotherapy

## Abstract

**Background:**

Lung adenocarcinoma (LUAD) has a very high morbidity and mortality rate, and its pathogenesis and treatment are still in the exploratory stage. Fatty acid metabolism plays a significant role in tumorigenesis, progression, and immune regulation. However, the gene expression of fatty acid metabolism in patients with LUAD and its relationship with prognosis remain unclear.

**Methods:**

We collected 309 fatty acid metabolism-related genes, established a LUAD risk model based on The Cancer Genome Atlas (TCGA) using Least Absolute Shrinkage Selection Operator (LASSO) regression analysis, and divided LUAD patients into high-risk and low-risk groups, which were further validated using the Gene Expression Omnibus (GEO) database. The nomogram, principal component analysis (PCA), and receiver operating characteristic (ROC) curves showed that the model had the best predictive performance. The ROC curves and calibration plots confirmed that the nomogram had good predictive power. We further analyzed the differences in clinical characteristics, immune cell infiltration, immune-related functions, chemotherapy drug sensitivity, and immunotherapy efficacy between the high-risk and low-risk groups. We also analyzed the enrichment pathways and protein–protein interaction (PPI) networks of different genes in the high-risk and low-risk groups to screen for target genes and further explored the correlation between target genes and differences in survival prognosis, clinical characteristics, gene mutations, and immune cells.

**Results:**

Risk score and staging are independent prognostic factors for patients with LUAD. The high-risk group had lower immune cell infiltration, was more sensitive to chemotherapeutic agents, and had a poorer survival prognosis. We also obtained three pivotal genes with poor survival prognosis in the high expression group, which were strongly associated with clinical symptoms and immune cells.

**Conclusion:**

Risk score and staging are independent prognostic factors for patients with LUAD. The high-risk group had lower immune cell infiltration, was more sensitive to chemotherapeutic agents, and had a poorer survival prognosis. We also obtained three survival prognosis-associated target genes that are closely associated with clinical symptoms and immune cells and may be potential targets for immune-targeted therapy in LUAD.

## Introduction

According to related reports, lung cancer ranks first in cancer-related death in 2020 (1). Lung adenocarcinoma (LUAD) is the most common subtype of lung cancer and has a higher incidence in women, accounting for 38.5% of all lung cancers (2). LUAD is microanatomically divided into two categories (3–5). Fatty acids are an important source of energy and a component of the cell structure in most species, including humans, and consist of carboxy-terminated and long-chain hydrocarbons. Previous studies have reported that abnormal fatty acid metabolism leads to a variety of diseases (6). A growing number of researchers have found that fatty acid metabolism plays an important role in the recognition, occurrence, and progression of various cancers, including but not limited to breast, prostate, ovarian, liver, and colon cancers (7–10). For example, dysregulation of fatty acid metabolism may interfere with the efficacy of chemotherapy and radiotherapy and immunotherapy in breast cancer patients (11). To date, fatty acid metabolism in LUAD has not been fully defined and more studies are needed in order to unravel this mystery.

We first screened fatty acid metabolism-related genes that were differentially expressed in tumor and normal samples, then screened survival prognosis-related genes, and finally constructed a prognostic risk model based on The Cancer Genome Atlas (TCGA) database. LUAD patients in the TCGA and Gene Expression Omnibus (GEO) databases were classified into high-risk and low-risk groups based on the median risk scores of samples in the TCGA database. The prognostic risk model was further validated using the public GEO database, and the fatty acid metabolic risk model for LUAD patients based on the TCGA database was constructed and validated from different perspectives. The differences between high-risk and low-risk LUAD patients in terms of immune cell infiltration, gene mutation, chemotherapeutic drug sensitivity, and immunotherapy effect were explored. Finally, we mapped the protein–protein interaction (PPI) network, on the basis of which the top 10 central genes were selected and the differences between network central genes and survival prognosis, clinical characteristics, and immune cells were further analyzed. In conclusion, our findings suggest that genes related to fatty acid metabolism may be potential prognostic markers for patients with LUAD and may become future therapeutic targets. The construction of risk scoring models has made it possible to individualize the treatment of LUAD patients.

## Materials and Methods

### Clinical Data Collection and Collation

We obtained transcription profiling data and clinical data for LUAD patients from the TCGA database (https://portal.gdc.cancer.gov/) (535 LUAD samples and 59 normal LUAD samples) and the GEO database (https://www.ncbi.nlm.nih.gov/geo/query/acc.cgi?acc= GSE11969) (GSE11969 and GPL7015) (94 LUAD samples). The clinical characteristics of the TCGA database are shown in **Supplementary Table 1**. The gene IDs of the samples were converted from the human gene annotation file to the corresponding gene symbols and averaged if multiple probes targeted the same gene ID. TCGA-LUAD is the test set, and GEO-LUAD is the train set.

### Acquisition of Genes Related to fatty Acid Metabolism

In combination with previous studies (9), we obtained three gene sets (KEGG fatty acid metabolism pathway, Reactome fatty acid metabolism genes, and Hallmark fatty acid metabolism genes) from Molecular Signature Database v7.2 (MSigDB). The screening yielded 309 genes related to fatty acid metabolism (**Supplementary Table 2**).

### Construction and Validation of a Fatty Acid Metabolic Risk Score Model

First, the “limma” R package was used to perform differential analysis to screen for differential genes related to fatty acid metabolism, and genes with LogFC < 0.585 and FDR < 0.05 were considered statistically significant. The “clusterprofiler” R package was used to enrich the GO and KEGG pathways of differential genes to determine their main biological features and cellular functional pathways. Differences were statistically significant when *p*-values and corrected *p*-values <0.05. Finally, the results of the enrichment analysis were visualized using the “ggplot 2” and “goplot” R packages. After removing patients with a survival time of less than 30 days, sequencing data of differentially expressed genes associated with fatty acid metabolism in the samples were combined with survival data, and genes associated with prognosis were screened from those associated with fatty acid metabolism by univariate Cox regression analysis based on the train set, and the cutoff point is set to *p*-value < 0.05. The correlation between the mutation frequency of genes in the train set samples and the mutated genes was analyzed using the “maftools” R package. The “Glmnet” R package was used for genes associated with the prognosis of fatty acid metabolism in LUAD. Based on the TCGA database, a prognostic risk score model for predicting OS in LUAD samples was developed using Least Absolute Shrinkage Selection Operator (LASSO) Cox regression analysis. The risk score formula was as follows.


$$risk\;score\;=\sum_{1}^{\text{i}}Coefi*ExpGenei$$

The “Coef” represents non-zero regression coefficients calculated using the LASSO Cox regression analysis (**Supplementary Table 3**), and “ExpGene” is the expression values of genes from the prognostic risk score model.

LUAD patients were divided into high-risk and low-risk groups according to the median fatty acid risk score of the TCGA-LUAD cohort sample, and the K-M method was used to analyze whether the high-risk and low-risk groups differed in terms of survival prognosis. The feasibility of the model was further validated using the GEO database. Nomograms, PCA, and ROC were used to ensure the accuracy of the model.

### PCA

PCA of gene expression profiles of fatty acid risk scoring models was performed using the “limma” R package for both train and test sets, including the expression profiles of differentially expressed genes associated with fatty acid metabolism in the train set. Fatty acid risk scoring models were constructed and the results were visualized using the “ggplot2” R package.

### Comprehensive Analysis of the Risk Scoring Model and Clinical Characteristics of LUAD Patients

Clinical information (stage) and fatty acid risk score of patients with LUAD were combined after excluding survival time of less than 30 days and missing data. Independent prognostic indicators were screened using univariate and multivariate Cox regression (*p*-value < 0.05).

### Construction and Evaluation of the Nomograms of LUAD Patients

To further investigate the overall survival (OS) of individual LUAD patients, a predictive model based on independent clinical parameters was developed using the “nomogram” R package. ROC curves and calibration plots were used to measure the ability of nomogram to predict prognosis.

### Characteristics of Patients in High- and Low-Risk Groups

Gene mutation data were downloaded from the TCGA database to calculate the tumor mutation burden (TMB) of LUAD patients. The “ggpubr” R package was used to explore whether there was an association between patient risk score and the frequency of tumor mutations in target genes. The immune cell infiltration file was downloaded from Timer2.0 (http://timer.cistrome.org/) to estimate the relationship between immune cell infiltration and risk score (for all TCGA tumors), using the “limma” and “pheatmap” R package for difference analysis, and the results were visualized. We further analyzed whether there were differences in immune-related functions between the high- and low-risk groups using the “GSVA” and “GSEABase” packages. The “PRRophetic” R package was used to predict the semi-inhibitory concentrations of cisplatin, gemcitabine, and paclitaxel in each sample, which indicates the effectiveness of a substance to inhibit a specific biological or biochemical function. The TIDE online database (http://tide.dfci.harvard.edu/) was used to predict the effect of immunotherapy in the high-risk and low-risk groups, and differences were considered statistically significant when the *p*-value < 0.05.

### Protein–Protein Interaction Network and Target Gene Characteristics

After screening for differential genes between high- and low-risk scoring groups, PPI network data [generated interaction score >0.90 (medium confidence)] were plotted online using the STRING online database (https://cn.string-db.org/). The 10 most pooled hub genes were screened using the cubHubba plugin of Cytoscape software (version: 3.9.1). Based on these 10 hub genes, the target genes differentially expressed in tumor tissues and normal tissues (cutoff value log2FC > 1, *p*-value < 0.05) and associated with postnatal survival were screened by the GEPIA (http://gepia.cancer-pku.cn) online database. The CIBERSORTx (https://cibersortx.stanford.edu/index.php online database was used to analyze the infiltration of 22 tumor-infiltrating lymphocyte-associated target genes in the microenvironment of high-risk and low-risk LUAD patients for significance ranking analysis 1,000 times, and “reshape2” and “ggpubr” R packages were used to visualize the difference results. The “limma” and “ggpubr” R packages were used to analyze the relationship between target gene expression and clinical characteristics (stage, T, N, M, age, and gender) of LUAD patients. Finally, the correlations between target genes were evaluated based on the GEPIA (http://gepia.cancer-pku.cn) online database and the Spearman test.

## Results

### Enrichment Analysis of Tumor and Normal Samples

**Figure 1:**
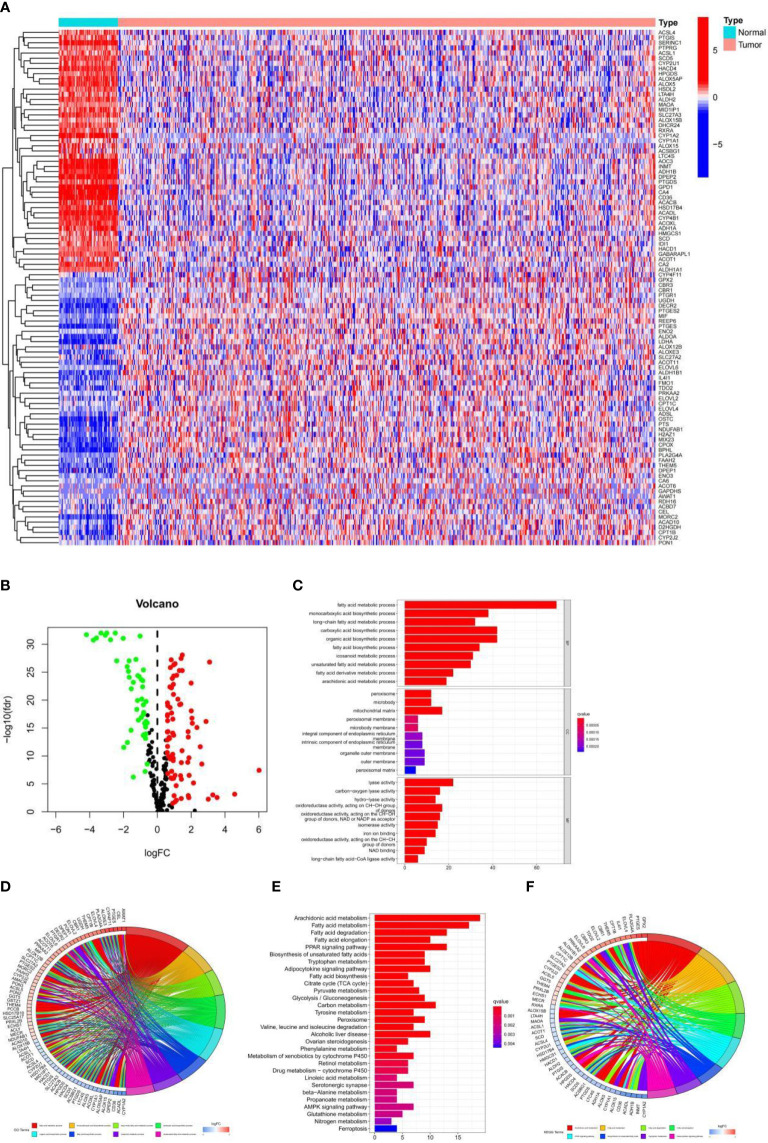
**(A)** Heat map of 126 genes in the TCGA-LUAD cohort. **(B)** Volcano map of the 126 fatty acid metabolism-related differential genes in the TCGA-LUAD cohort. **(C, D)** GO analysis of fatty acid metabolism-related differential genes in the TCGA-LUAD cohort. **(E, F)** KEGG analysis of fatty acid metabolism-related differential genes in the TCGA-LUAD cohort.

By comparing the expression levels of fatty acid metabolism-related genes in tumor and normal samples in the TCGA database, 126 genes were screened in the TCGA-LUAD cohort (*p*-value < 0.05, FDR < 0.585), of which 79 genes were upregulated in tumor tissue samples and 47 genes were downregulated in tumor tissue samples. Heat maps and volcano maps of differentially expressed genes in normal and tumor samples are shown in **Figures 1A, B**. We know from GO enrichment analysis that among the biological processes, fatty acid metabolic processes, long-chain fatty acid metabolic processes, and fatty acid biosynthesis processes are highly enriched terms (**Figure 1C**), and **Figure 1D** shows the 72 most significantly enriched genes and enriched pathways. The results of KEGG enrichment analysis of genes and genomes in Kyoto showed that Fatty acid metabolism, Fatty acid degradation, Fatty acid elongation, and Fatty acid biosynthesis were all highly enriched KEGG items (**Figure 1E**), and **Figure 1F** shows the 55 most significantly enriched genes and enriched pathways. These results suggest that fatty acid metabolism-related genes in LUAD are clustered in biological pathways related to fatty acid metabolism and are closely related to fatty acid metabolism.

### Construction of Risk Scoring Model in the Train Set

**Figure 2:**
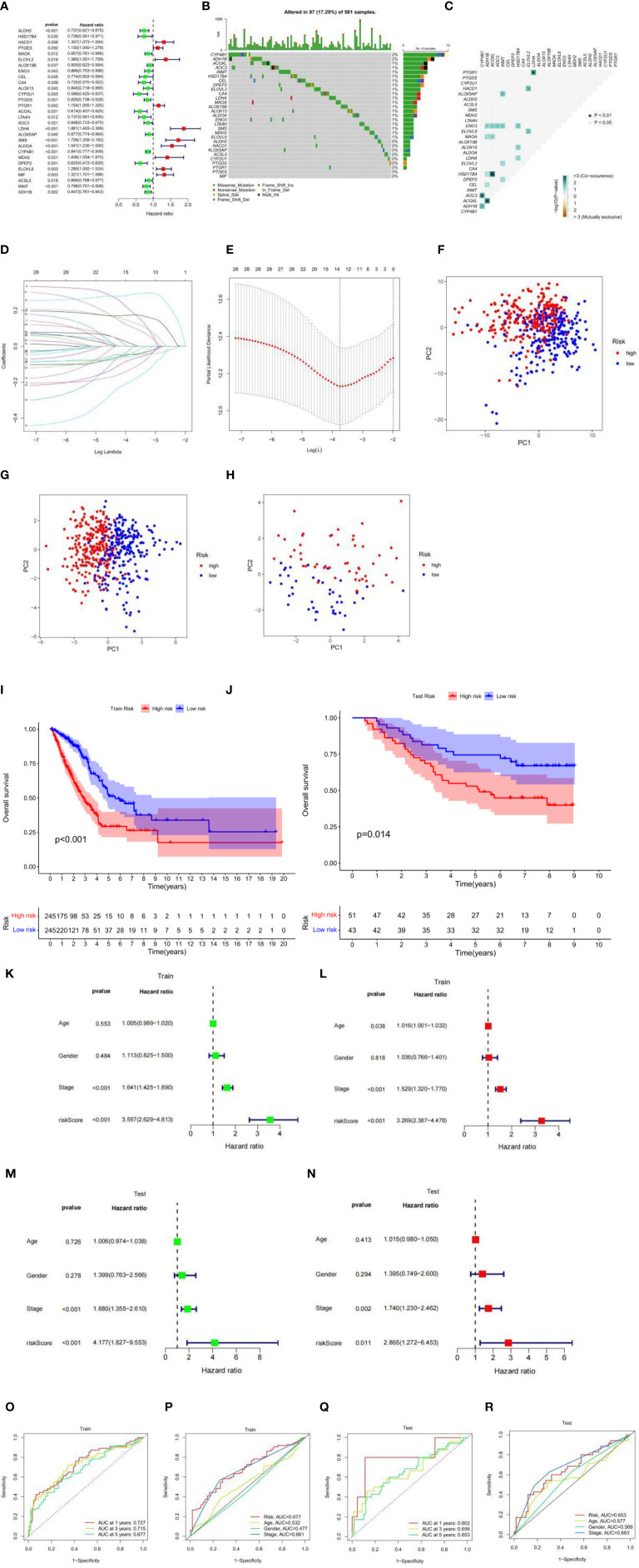
**(A)** Univariate analysis of genes related to fatty acid metabolism. When the hazard ratio of a gene is >1, it indicates that the gene is a risk factor for the corresponding tumor, and vice versa. **(B)** Gene mutations in patients with TCGA-LUAD. **(C)** Correlation of mutations in 29 fatty acid metabolism genes. Brown color indicates negative correlation, and blue color indicates positive correlation. p < 0.05, *p < 0.01. **(D)** LASSO coefficient spectrum of 29 fatty acid metabolism genes. **(E)** Cross-validation of adjustment parameter selection in a proportional hazards model. **(F)** PCA based on all fatty acid metabolism-related genes in the TCGA-LUAD cohort. **(G)** PCA based on fatty acid metabolism risk scores in the TCGA-LUAD cohort. **(H)** PCA based on fatty acid metabolism risk scores in the GEO-LUAD cohort. The red group represents high-risk patients, and the blue group represents low-risk patients. **(I)** OS by fatty acid risk score in the TCGA-LUAD cohort. **(J)** OS by fatty acid risk score in the GEO-LUAD cohort. **(K)** Results of univariate Cox analysis in the TCGA-LUAD cohort. **(L)** Multivariate Cox analysis results in the TCGA-LUAD cohort. **(M)** Results of univariate Cox analysis in the GEO-LUAD cohort. **(N)** Multivariate Cox analysis results in the GEO-LUAD cohort. **(O)** AUC values at 1, 3, and 5 years in the TCGA-LUAD cohort. **(P)** ROC curves of risk scores and clinical characteristics in the TCGA-LUAD cohort. **(Q)** AUC values at 1, 3, and 5 years in the GEO-LUAD cohort. **(R)** ROC curves of risk scores and clinical characteristics in the GEO-LUAD cohort.

The TCGA-LUAD cohort was used as a train set. A total of 126 cases of fatty acid metabolism-related genes were screened from the TCGA-LUAD cohort. After excluding data from patients with a survival time of less than 30 days and null values, 29 fatty acid metabolism-related genes associated with patient survival were screened using univariate Cox analysis (**Figure 2A**). The somatic mutation profiles of the 29 prognosis-related genes showed a mutation frequency of 17.29% in the 561 LUAD samples (a total of 97 cases were mutated) (**Figure 2B**), with ADH1B and CYP4B1 having the highest mutation frequencies, followed by ELOVL6, MDH2, SMS, LTA4H, ENO3, ALDOA, ALOX15, ALOX15B, MAOA, LDHA, CA4, ELOVL2, DPEP2, CEL, HSD17B4, INMT, AOC3, and ACOXL, and no mutations in other genes. Further analysis revealed a mutational positive relationship between ACOXL and HSD17B4, ADH1B; CYP4B1 and AOC3, and PTGR1 and LDHA (**Figure 2C**). These 29 genes were further incorporated into the LASSO logistic regression algorithm based on the TCGA-LUAD cohort. A total of 14 genes for constructing fatty acid risk score models were obtained by LASSO Cox regression analysis (**Figures 2D, E**), namely, ALDH2, HACD1, ELOVL2, ENO3, CEL, CA4, CYP2U1, LDHA, ALOX5AP, SMS, ALDOA, CYP4B1, DPEP2, and ELOVL6.

Using this risk score model, the LUAD samples (low and high risk) were completely distinguished (**Figures 2F, G**), while the GEO-LUAD cohort was similarly distinguished as a test set (**Figure 2H**). In this fatty acid risk model, the median risk score of the TCGA-LUAD cohort was used as the cutoff value, and 490 TCGA-LUAD patients were classified into a high-risk group (n = 245) and a low-risk group (n = 245), and 94 GEO-LUAD patients were classified into a high-risk group (n = 51) and a low-risk group (n = 43). In both the test and train groups, the low-risk group had a better clinical prognosis (p-value < 0.05) (**Figures 2I, J**). Univariate prognostic COX analyses of the train and test sets showed that stage and risk score were independent prognostic factors, and multivariate COX analyses also showed this result (p-value < 0.05) (**Figures 2K–N**). Using the ROC curve estimation model to evaluate the reliability of the fatty acid risk score, the area under the curve (AUC) was 0.727, 0.715, and 0.677 for the train sets at 1, 3, and 5 years, respectively (**Figure 2O**). The AUC was 0.802, 0.656, and 0.653 for the test sets at 1, 3, and 5 years, respectively (**Figure 2Q**); the train set risk model has the largest area under the ROC curve (**Figure 2P**) and the test set risk model. The ROC curve was second only to stage, which proved the most reliable model of fatty acid metabolism risk (**Figure 2R**).

### Construction and Evaluation of Nomograph

**Figure 3:**
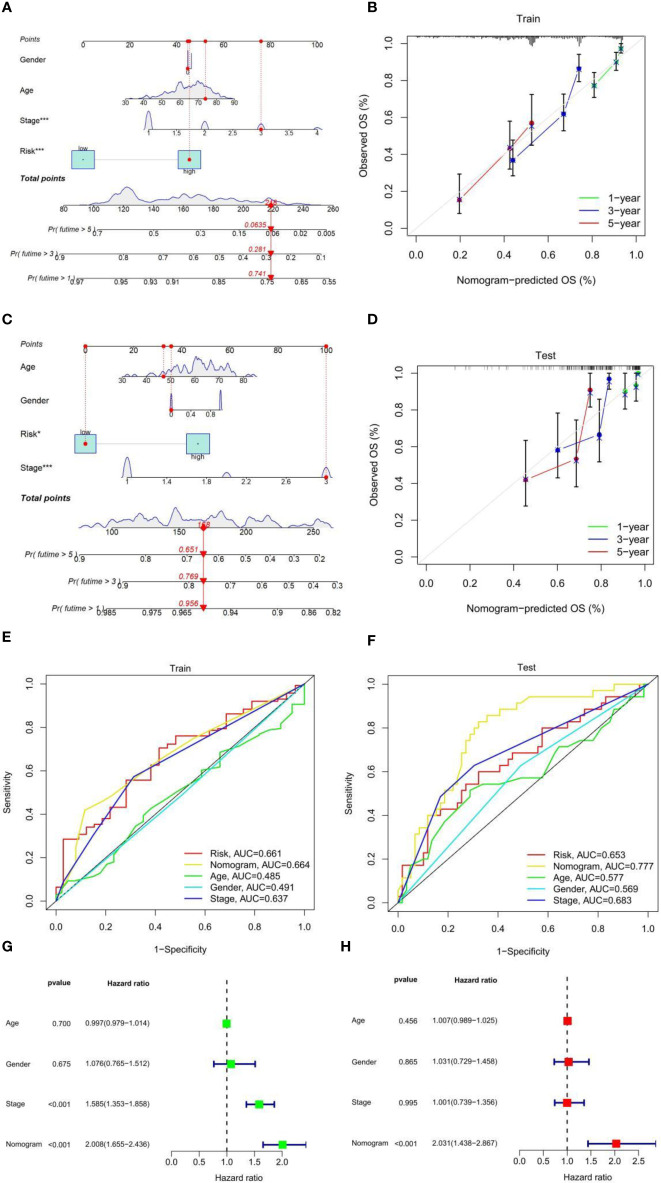
**(A)** Nomogram to predict 1-, 3-, and 5-year OS in the TCGA-LUAD cohort. **(B)** Calibration plot to assess the accuracy of a nomogram to predict 1-, 3-, and 5-year OS in the TCGA-LUAD cohort. **(C)** Nomogram to predict 1-, 3-, and 5-year OS in the GEO-LUAD cohort. **(D)** Calibration plot to assess the accuracy of a nomogram to predict 1-, 3-, and 5-year OS in the GEO-LUAD cohort. **(E)** ROC curves of risk score and clinical characteristics in the TCGA-LUAD cohort. **(F)** ROC curves of risk score and clinical characteristics in the GEO-LUAD cohort. **(G)** Univariate Cox analysis. **(H)** Multivariate Cox analysis.

Prognostic factors such as risk score, stage, age, and sex were included in the nomogram to assess the predictive ability of individual OS, and to calculate the prediction effect of the nomogram on 1-, 3-, and 5-year OS in patients with LUAD (**Figure 3A**). The nomogram was applied to patients in the test set (GEO database), the effect was good (**Figure 3C**), and the calibration charts were close to the ideal curve (**Figures 3B, D**). In addition, ROC curve analysis is carried out to verify the practicability of the nomogram, and the AUC of train set and test set risk, nomogram, age, gender, and the stage is calculated (**Figures 3E,F**). Nomogram AUC is the largest, indicating that the nomogram has the best prediction effect. Further univariate and multivariate Cox analysis of the train set data showed that the classification and risk score were independent prognostic factors (p-value < 0.001) (**Figures 3G, H**). In conclusion, the prognostic ability of this fatty acid prognostic model has been validated from several perspectives.

### Immune-Related Characteristic and Chemical Response in the Low- and High-Risk Score Groups

**Figure 4:**
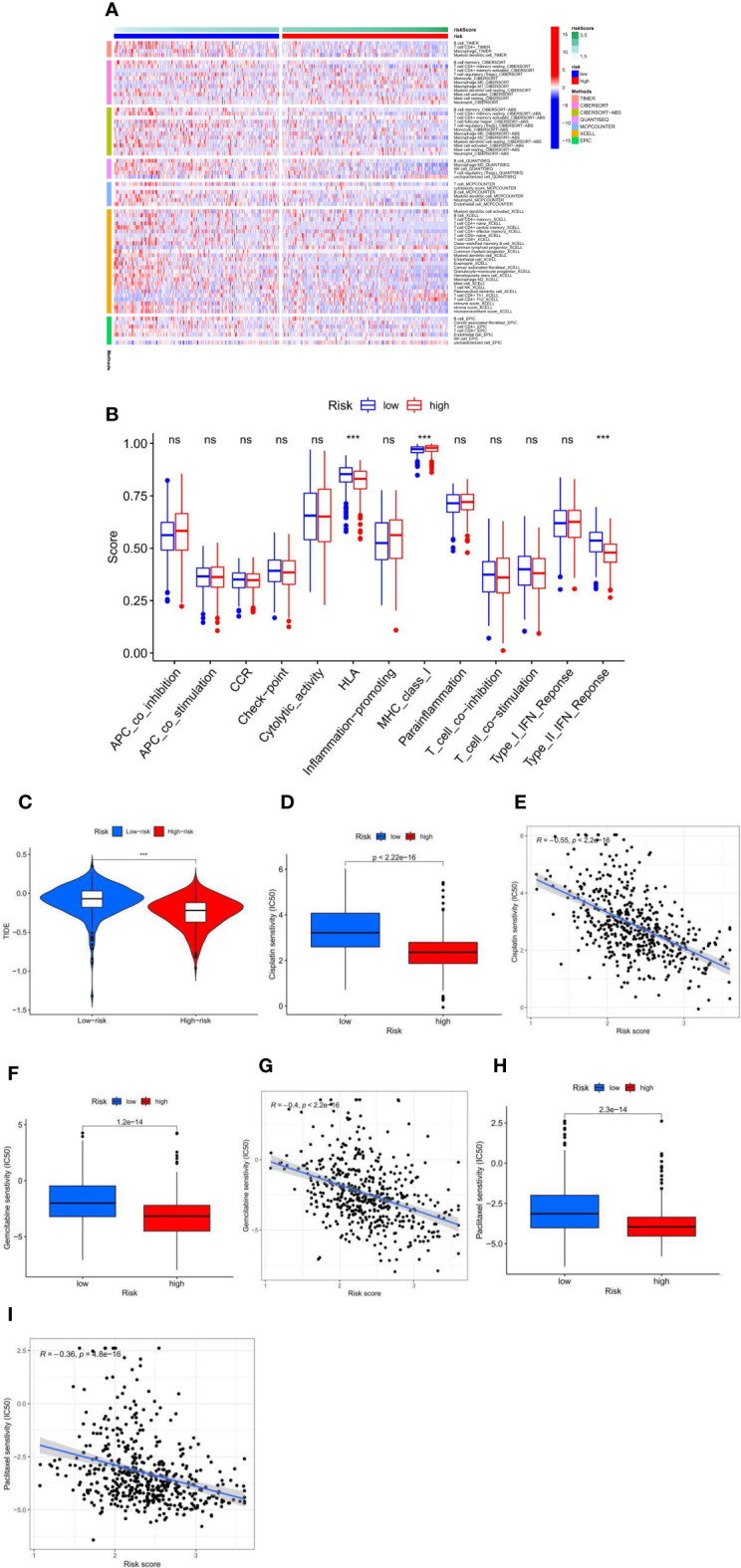
**(A)** The immune infiltration of immune cell types in high-risk and low-risk patients in the TCGA-LUAD cohort. **(B)** Analysis of immune function in high-risk and low-risk patients in the TCGA-LUAD cohort. ***p < 0.001, ns p > 0.05. **(C)** High-risk and low-risk LUAD patients with TIDE scores in the TCGA-LUAD cohort. **(D–I)** Fatty acid metabolism score and cisplatin **(D, E)**, gemcitabine **(F, G)**, and paclitaxel **(H, I)** chemotherapeutic drug sensitivity analysis in the TCGA-LUAD cohort.

Immune cell infiltration showed immunological differences between the high-risk and low-risk groups, with significantly increased abundance of Macrophage M0, Macrophage M1, T cell CD4+ Th1, T cell CD4+ Th2, and T cell CD4+ memory activated in the high-risk group and B cell memory, Macrophage M2, T cell CD4+ central memory, T cell CD4+ effector memory, T cell CD8+, Myeloid dendritic cell resting, Myeloid dendritic cell activated, and T cell regulatory significantly increased in the low-risk group (**Figure 4A**). Immune function analysis (**Figure 4B**) showed that HLA and Type_II_IFN_Response immune-related functions were active in the low-risk group and MHC_class_I immune-related functions were active in the high-risk group. TIDE scores were lower in the high-risk group than in the low-risk group (**Figure 4C**), which proved that the high-risk group had a better effect of immunotherapy. In the risk score and chemical drug sensitivity analysis, we found that the risk score was negatively correlated with cisplatin, gemcitabine, and paclitaxel chemotherapy drug sensitivity. The IC50 of patients in the high-risk group with the lower value (**Figures 4D–I**) proves that patients in the high-risk group were more sensitive to chemotherapy drugs than those in the low-risk group. In conclusion, the quantification of fatty acid metabolic risk scores is of great importance in patients with LUAD, not only to assess the prognosis of immunotherapy, but also to evaluate the effect of chemotherapy, which may be a new biomarker to change the outcome of treatment in patients with LUAD.

### PPI Network of Differentially Expressed Genes in Low- and High-Risk Groups

**Figure 5:**
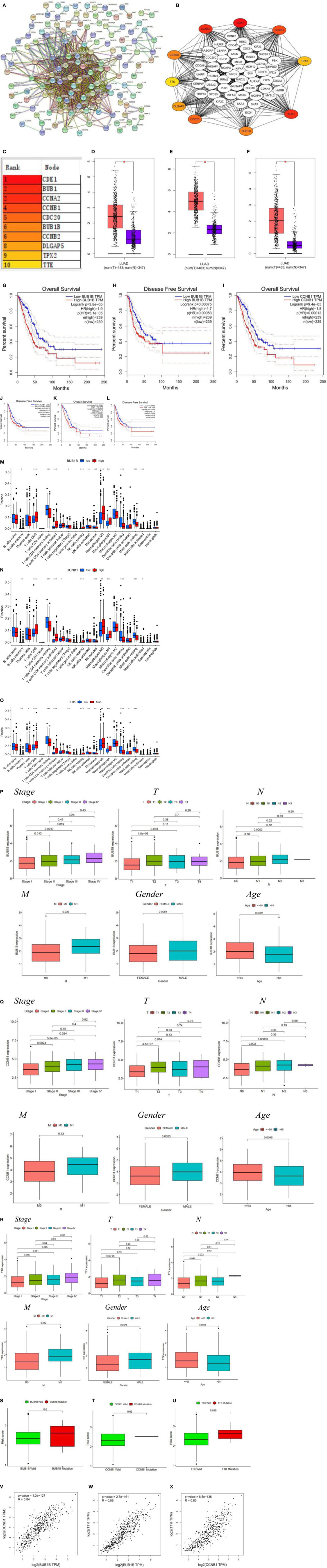
**(A)** PPI network of risk differential genes. **(B, C)** Top 10 hub genes of the gene expression network. **(D–F)** Comparison of BUB1B **(D)**, CCNB1 **(E)**, and TTK **(F)** mRNA expression levels of each gene in LUAD tissue and normal lung tissue. The expression levels of all genes in cancer tissues were higher than those in normal lung tissues. The red and blue boxes represent tumor and normal tissue, respectively. Red asterisks indicate significant differences in the expression of each mRNA (p-value < 0.05). **(G–L)** OS and RFS of 3 target genes of BUB1B **(G, H)**, CCNB1 **(I, J)**, and TTK **(K, L)**. **(M–O)** Immune cell content of 22 immune cell types in BUB1B **(M)**, CCNB1 **(N)**, and TTK **(O)** Hub genes. **(P–R)** The relationship of BUB1B **(P)**, CCNB1 **(Q)**, and TTK **(R)** target genes and clinicopathological features, including TNM stage (Stage), tumor invasion **(T)**, lymphoid metastasis (N), distal metastasis **(M)**, gender (Gender), and age (Age). **(S–U)** Correlation analysis between the type of gene mutation (mutant, wild type) and risk score, BUB1B **(S)**, CCNB1 **(T)**, and TTK **(U)**. **(V)** BUB1B and CCNB1 correlation analysis. **(W)** BUB1B and TTK correlation analysis. **(X)** CCNB1 and TTK correlation analysis. *p < 0.05, **p < 0.01, ***p < 0.001.

Differential gene interactions between low- and high-risk groups were analyzed using the STRING online database. The differential gene PPI network is shown in **Figure 5A**. Hub genes were identified from differential genes using the Cytoscape plugin cytoHubba. We chose a total of 10 genes in the network (**Figure 5B**) and ranked CDK1, BUB1, CCNA2, CCNB1, CDC20, BUB1B, CCNB2, DLGAP5, TPX2, and TTK utilizing the degree method **Figure 5C**). Through differential analysis and survival analysis, three hub genes were screened as target genes. We performed a differential analysis based on TCGA and GTEx databases using GEPIA and found that BUB1B (**Figure 5D**), CCNB1 (**Figure 5E**), and TTK (**Figure 5F**) were significantly overexpressed in LUAD tissues compared with normal samples (p-value < 0.05). BUB1B (**Figures 5G, H**), CCNB1 (**Figures 5I, J**), and TTK (**Figures 5K, L**) were significantly associated with survival prognosis (OS and RFS) (p-value < 0.01), and the higher the gene expression, the worse the prognosis.

The results of immune cell infiltration showed that the upregulated immune cells in the gene BUB1B high expression group were T cells CD8, T cells CD4 memory activated, NK cells resting, Macrophages M0, and Macrophages M1, and the downregulated immune cells were B cells naive, T cells CD4 memory resting, T cells regulatory (Tregs), NK cells activated, Monocytes, Dendritic cells resting, and Mast cells resting (**Figure 5M**); the upregulated immune cells in the CCNB1 high expression group were T cells CD8, T cells CD4 memory activated, T cells follicular helper, NK cells resting, Macrophages M0, and Macrophages M1, and the downregulated immune cells were B cells memory, T cells CD4 memory resting, Monocytes, Dendritic cells resting, and Mast cells resting (**Figure 5N**); the upregulated immune cells in the TTK high expression group were T cells CD8, T cells CD4 memory activated, Macrophages M0, Macrophages M1, and Dendritic cells activated, and downregulated immune cells were B cells memory, Plasma cells, T cells CD4 memory resting, T cells regulatory (Tregs), NK cells activated, Monocytes, Dendritic cells resting, and Mast cells resting (**Figure 5O**). We noted that the infiltration of activated immune cells was abundant and active in the high expression group, which was suitable for immunotherapy. Clinical correlation analysis showed that in the BUB1B, CCNB1, and TTK genes, stage I patients had significantly lower expression levels than stage II, III, and IV patients, and higher gene expression levels in men and patients younger than 65 years (p-value < 0.05) (**Figures 5P–R**). There was no significant difference between BUB1B, CCNB1 mutant, and wild-type risk scores (**Figures 5S, T**), and there was a significant difference between TTK mutant and wild-type risk scores (p-value < 0.05) ( **Figure 5U**). Based on the GEPIA (http://gepia.cancer-pku.cn) online database, correlations between genes were analyzed using the Spearman test. The three genes BUB1B, CCNB1, and TTK were positively correlated with each other with statistically significant differences (**Figures 5V–X**) (p-value < 0.001).

## Discussion

Lung cancer is one of the most common cancers worldwide (11.6% of all cases) and is the leading cause of cancer-related deaths (18.4% of all cancer-related deaths) (12). Despite significant improvements in early detection, targeted therapies, and chemotherapy techniques over the past decades, the OS of patients with LUAD remains low. Therefore, there is an urgent need for complete clarity on the principles of LUAD pathogenesis and development, and more potential targets of clinical therapeutic benefit need to be identified. Cellular metabolism is critical for cell survival and development, and tumor cells suffer from abnormal cellular metabolism due to loss of function of tumor suppressor genes or activation of oncogenes. Changes in metabolism are widely observed in various cancer cells (13) and are used in tumor therapy (14–16). Disturbances in fatty acid metabolism are associated with tumor progression, and fatty acid metabolism has been applied to explore the progression of various cancers, such as colon and breast cancer, and to describe the efficacy of therapeutic and prognostic interventions (17, 18). Exploring the role of different fatty acid metabolic patterns in LUAD can help to understand the role of fatty acid metabolism in LUAD progression and thus guide effective therapeutic strategies. Although there have been many prediction models for studies on LUAD, with very few on fatty acid metabolism, we selected two different databases, TCGA and GEO, set up a train set and a test set, and used the test set to verify the accuracy of the train set, partially compensating for the lack of clinical trials, and constructed individual patient risk prediction models that can accurately predict the treatment outcome of individual patients. We further analyzed the relationship between target genes and immune cell infiltration and clinical characteristics.

In our study, we found that genes related to fatty acid metabolism were strongly associated with LUAD patients, especially in terms of OS. A fatty acid metabolism risk model was established based on the TCGA-LUAD cohort. The TCGA-LUAD cohort was the train set and the GEO-LUAD cohort was the test set. According to the TCGA-LUAD cohort, patients with reduced median risk scores were divided into a high-risk group and a low-risk group. Univariate and multivariate Cox analyses showed that risk scores and stage were independent of other factors in predicting clinical survival in patients with TCGA-LUAD and could be used as independent prognostic indicators. The GEO test set verifies this result well. We also constructed nomograms that provide a good assessment of each patient’s clinical survival. Immunotherapy aims to activate the natural immune molecular components of the tumor microenvironment to defend against cancer. Numerous studies have reported that the main antitumor features of the tumor microenvironment are CD8+ cytotoxic T cells, Th1 helper cells, and their associated cytokines, such as interferons (IFNs) (19). High expression of Th1, CD8+ T, and effector memory T cells has been shown to be associated with better prognosis (20). Recent findings by Ferreira et al. found that Tregs produce more effective anti-tumor immunity by providing the necessary cytokines (21). Wu et al. suggested that T-cell CD4+ central memory inhibits lymph node metastasis, thereby improving the prognosis of patients with oral squamous cell carcinoma (22). Macrophages are key factors in LUAD metastasis, with the M2 subtype stimulating lung cancer cell invasion and the M1 subtype inhibiting tumor formation (23). In our study, the low-risk group was enriched in T-cell regulatory cells (Tregs), T-cell CD8+, T-cell CD4+ central memory, and T-cell CD4+ effector memory, consistent with the survival advantage of the low-risk group. We also noted that the high-risk group had lower dendritic cell content than the low-risk group. Dendritic cells play a crucial role in the initiation of antigen-specific immunity (24) and present antigens to T cells, promoting the antitumor activity of CD4+ and CD8+ T cells through cell–cell contact and in cytokine release activity (25). From this, we hypothesized that lower levels of dendritic cells in the high-risk group may be associated with survival disadvantage, which points us to the next direction of research, namely, by stimulation of dendritic cells to activate immune responses and enhance immunotherapy and other therapeutic options to kill tumor cells. Type II interferon (IFN) response activation in the low-risk group suggests that patients in the immunosuppressed low-risk group should also respond to immunotherapy and immunosuppressive factors such as TGF-β in the low-risk group and that TGF-β inhibitors combined with monoclonal antibodies would be a good therapeutic option.

The risk score and chemical drug sensitivity analysis showed that the risk score was sensitive to three common chemotherapy drugs in LUAD, namely, cisplatin, gemcitabine, and paclitaxel, and was negatively correlated, which opened up a new way for further guiding clinical treatment of lung adenocarcinoma. Based on the differentially expressed genes between high-risk and low-risk groups, we drew the PPI network and further screened out 10 hub genes. These 10 hub genes were all significantly different between tumor groups and normal samples (*p*-value < 0.01); OS and disease-free survival (DFS) analysis showed that there were significant differences in BUB1B, CCNB1, and TTK (*p*-value < 0.01). These three genes were highly expressed in tumors and were high-risk genes. The higher the gene expression, the worse the prognosis. BUB1B is not only a key component of the spindle assembly checkpoint, but its abnormal expression usually represents a poor prognosis of the tumor (26). A present meta-analysis showed that high BUB1B expression predicts poor OS and progression-free survival (PFS) and that BUB1B is an important biomarker for poor prognosis and poor clinicopathological outcome in patients with LUAD (27). Zhou et al. (28) found that BUB1B was upregulated in LUAD, and clinical survival is shorter in LUAD patients with high BUB1B expression. Expression of CCNB1 is significantly elevated in samples from LUAD patients and is associated with advanced tumor stage and shorter OS (29), and TTK is a mitotic checkpoint kinase that is present in higher amounts in some human cancers than in normal tissue (30). In addition, high expression of TTK was positively correlated with higher invasiveness and treatment resistance of breast cancer, suggesting that TTK may be involved in cancer cell proliferation and poor patient survival, and is an independent prognostic factor (31). The high-risk score of TTK mutation type indicates that this gene is prone to a gene mutation in LUAD and may be a potential gene mutation therapy target. These findings are consistent with our findings, validating the accuracy of our study and again validating the scientificity of the model. These three hub gene activation states have more infiltration and more active immune cells, suggesting that immunotherapy may change the survival of patients with poor prognoses. However, although we obtained that BUB1B, CCNB1, and TTK are positively correlated in LUAD, there is no experimental confirmation yet, and further investigation is needed to investigate the intrinsic association.

## Conclusion

In conclusion, we generated a fatty acid metabolism risk model in patients with LUAD and showed that the fatty acid metabolism risk score was associated with immune cell infiltration, and chemotherapeutic and immunotherapy effects in LUAD patients. Three fatty acid metabolism genes not only are significantly associated with clinical staging and prognosis of LUAD patients, but also have great importance in immune cell infiltration. These three genes could be used as biomarkers for individualized treatment of LUAD patients and improve the prognosis of LUAD patients.

## Data Availability Statement

This study relied on publicly available data.The Cancer Genome Atlas (TCGA) and Gene Expression Omnibus(GEO) databases contain this information. The accession number(s) can be found in the article/**Supplementary Material**.

## Ethics Statement

The TCGA, GEO, Timer2.0, TIDE, STRING, CIBERSORTx, and GEPIA are public databases. Ethical approval has been granted to the patients who are part of the databases. Users can get relevant data for free and produce articles based on it. Because our research relies on open-source data, there are no ethical concerns or potential conflicts of interest.

## Author Contributions

DH and GX were in charge of the concept and design. GX provides administrative support. DH is in charge of data collection and assembly. DH did the data analysis and interpretation. All authors contributed to the article and approved the submitted version.

## Funding

This project is funded by the Scientific Research Project of the National Natural Science Foundation of China Commission (Project name: The mechanism of moduli TGF-induced EMT in NSCLC cells by the Circ-PTK2/miR-429/TIF1 γ axis; Approval No. 81872343).

## Conflict of Interest

The authors declare that the research was conducted in the absence of any commercial or financial relationships that could be construed as a potential conflict of interest.

## Publisher’s Note

All claims expressed in this article are solely those of the authors and do not necessarily represent those of their affiliated organizations, or those of the publisher, the editors and the reviewers. Any product that may be evaluated in this article, or claim that may be made by its manufacturer, is not guaranteed or endorsed by the publisher.

## Supplementary Material

Supplementary Table 1 Clinical characteristics of the TCGA data.

Supplementary Table 2 309 fatty acid metabolism-related genes were obtained from previous studies and GSEA.

Supplementary Table 3 Non-zero regression coefficients calculated using the LASSO Cox regression analysis.
