# Supplementary material for: Characteristics of Fatty Acid Metabolism in Lung Adenocarcinoma to Guide Clinical Treatment
Source: Front Immunol. 2022 Jul 1;13:916284. doi: 10.3389/fimmu.2022.916284 (PMC9289740; doi:10.3389/fimmu.2022.916284)
Supplement: Supplementary Table 1 — Clinical characteristics of the TCGA data. [file Table_1.doc]

TCGA clinical data

| Id | futime | fustat | Age | Gender | Grade | Stage | T | M | N |
| --- | --- | --- | --- | --- | --- | --- | --- | --- | --- |
| TCGA-J2-8192 | 739 | 0 | 65 | FEMALE | unknow | Stage IIA | T2a | MX | N1 |
| TCGA-91-8499 | 36 | 0 | 76 | FEMALE | unknow | Stage IA | T1b | MX | N0 |
| TCGA-53-7624 | 1043 | 1 | 40 | FEMALE | unknow | Stage IV | T2 | M1 | N0 |
| TCGA-55-6986 | 3261 | 0 | 74 | FEMALE | unknow | Stage IB | T2 | M0 | N0 |
| TCGA-38-4632 | 1357 | 1 | 42 | MALE | unknow | Stage IV | T2 | M1 | N1 |
| TCGA-78-7146 | 173 | 1 | 71 | FEMALE | unknow | Stage IIIA | T2 | M0 | N2 |
| TCGA-NJ-A4YG | 2261 | 0 | 65 | MALE | unknow | Stage IB | T2 | M0 | N0 |
| TCGA-86-8279 | 949 | 0 | 46 | MALE | unknow | Stage IIA | T2a | M0 | N1 |
| TCGA-50-6591 | 119 | 1 | 63 | FEMALE | unknow | Stage IV | T2 | M1 | N0 |
| TCGA-50-5939 | 460 | 1 | 85 | MALE | unknow | Stage IB | T2 | M0 | N0 |
| TCGA-62-8402 | 1498 | 1 | 73 | FEMALE | unknow | Stage IIIA | T2 | M0 | N2 |
| TCGA-49-4506 | 999 | 1 | 68 | FEMALE | unknow | Stage IIB | T2 | M0 | N1 |
| TCGA-91-6847 | 842 | 0 | 62 | FEMALE | unknow | Stage IB | T2 | MX | N0 |
| TCGA-97-8547 | 657 | 0 | 78 | FEMALE | unknow | Stage IIIA | T2a | MX | N2 |
| TCGA-97-7546 | 1285 | 0 | 76 | FEMALE | unknow | Stage IA | T1 | MX | N0 |
| TCGA-73-4659 | 711 | 1 | 66 | MALE | unknow | Stage IIIA | T2 | M0 | N2 |
| TCGA-55-A48Y | 630 | 0 | 69 | MALE | unknow | Stage IIA | T2b | M0 | N0 |
| TCGA-50-5049 | 3094 | 0 | 70 | MALE | unknow | Stage IA | T2 | M0 | N0 |
| TCGA-44-3396 | 1130 | 0 | 74 | FEMALE | unknow | Stage IIIA | T2 | M0 | N2 |
| TCGA-05-4405 | 610 | 0 | 74 | FEMALE | unknow | Stage IB | T2 | M0 | N0 |
| TCGA-78-8662 | 3361 | 1 | 53 | FEMALE | unknow | Stage IB | T2 | M0 | N0 |
| TCGA-50-8460 | 829 | 0 | 74 | MALE | unknow | Stage IA | T1a | M0 | N0 |
| TCGA-55-8087 | 462 | 0 | 59 | FEMALE | unknow | Stage IB | T2a | MX | N0 |
| TCGA-MP-A4TA | 950 | 1 | 75 | FEMALE | unknow | Stage IA | T1 | M0 | N0 |
| TCGA-55-7815 | 773 | 0 | 76 | MALE | unknow | Stage IB | T2a | MX | N0 |
| TCGA-55-8096 | 719 | 1 | 67 | FEMALE | unknow | Stage IB | T2a | MX | N0 |
| TCGA-NJ-A55R | 603 | 0 | 67 | MALE | unknow | Stage IA | T1b | MX | N0 |
| TCGA-91-7771 | 492 | 0 | 62 | MALE | unknow | Stage IIB | T3 | MX | N0 |
| TCGA-69-7760 | 202 | 0 | 73 | MALE | unknow | Stage IIB | T3 | M0 | N0 |
| TCGA-44-5644 | 863 | 0 | 51 | FEMALE | unknow | Stage IB | T2a | unknow | N0 |
| TCGA-69-7973 | 230 | 0 | 42 | FEMALE | unknow | Stage IB | T2a | M0 | N0 |
| TCGA-44-6145 | 595 | 0 | 62 | FEMALE | unknow | Stage IA | T1 | M0 | N0 |
| TCGA-64-1676 | 1728 | 0 | 58 | MALE | unknow | Stage IA | T1a | M0 | N0 |
| TCGA-97-7553 | 1870 | 0 | 58 | FEMALE | unknow | Stage IA | T1 | MX | N0 |
| TCGA-55-8511 | 552 | 0 | 73 | FEMALE | unknow | Stage IB | T2a | MX | N0 |
| TCGA-MP-A4TF | 336 | 1 | 58 | FEMALE | unknow | Stage IIA | T2b | M0 | N0 |
| TCGA-55-8204 | 515 | 0 | 87 | FEMALE | unknow | Stage IB | T2a | MX | N0 |
| TCGA-55-8616 | 48 | 0 | 58 | FEMALE | unknow | Stage IB | T2a | M0 | N0 |
| TCGA-86-8281 | 0 | 0 | 75 | MALE | unknow | Stage IA | T1 | M0 | NX |
| TCGA-55-7574 | 995 | 1 | 64 | FEMALE | unknow | Stage IB | T2a | M0 | N0 |
| TCGA-L9-A743 | 664 | 0 | 56 | MALE | unknow | Stage IIA | T2a | M0 | N1 |
| TCGA-73-4670 | 131 | 0 | 69 | FEMALE | unknow | Stage IV | T2 | M1 | N0 |
| TCGA-50-5066 | 1442 | 0 | 72 | MALE | unknow | Stage IB | T2 | M0 | N0 |
| TCGA-75-5126 |  | 0 | unknow | FEMALE | unknow | Stage IIIA | T3 | M0 | N2 |
| TCGA-64-5779 | 864 | 0 | 61 | MALE | unknow | Stage IIIA | T2 | M0 | N2 |
| TCGA-55-6543 | 435 | 0 | 60 | FEMALE | unknow | Stage IA | T1b | MX | N0 |
| TCGA-55-A48Z | 651 | 0 | 60 | FEMALE | unknow | Stage IIIB | T1a | MX | N3 |
| TCGA-86-8278 | 944 | 0 | 63 | FEMALE | unknow | Stage IIB | T2 | M0 | N1 |
| TCGA-L4-A4E5 | 578 | 0 | 48 | FEMALE | unknow | Stage I | T1 | M0 | N0 |
| TCGA-49-4505 | 428 | 1 | 61 | FEMALE | unknow | Stage IIB | T2 | M0 | N1 |
| TCGA-69-7980 | 411 | 0 | 70 | FEMALE | unknow | Stage I | T1b | M0 | N0 |
| TCGA-55-7725 | 442 | 0 | 68 | FEMALE | unknow | Stage IA | T1a | MX | N0 |
| TCGA-78-7162 | 3169 | 1 | 75 | MALE | unknow | Stage IA | T1 | M0 | N0 |
| TCGA-80-5608 | 2832 | 0 | unknow | FEMALE | unknow | Stage IA | T1 | M0 | N0 |
| TCGA-78-7159 | 1974 | 0 | 60 | FEMALE | unknow | Stage IA | T1 | M0 | NX |
| TCGA-MP-A4TI | 429 | 1 | 72 | MALE | unknow | Stage IIA | T2a | M0 | N1 |
| TCGA-78-7542 | 321 | 1 | 56 | MALE | unknow | Stage IB | T2 | M0 | N0 |
| TCGA-91-6836 | 417 | 0 | 52 | FEMALE | unknow | Stage IB | T2 | MX | N0 |
| TCGA-78-7537 | 1622 | 1 | 72 | MALE | unknow | Stage IB | T2 | M0 | N0 |
| TCGA-97-7941 | 484 | 0 | 72 | FEMALE | unknow | Stage IA | T1b | MX | N0 |
| TCGA-MP-A4TJ | 339 | 1 | 62 | FEMALE | unknow | Stage IA | T1 | M0 | N0 |
| TCGA-78-8660 | 321 | 1 | 69 | MALE | unknow | Stage IIB | T2 | M0 | N1 |
| TCGA-49-AARQ | 6732 | 0 | 41 | FEMALE | unknow | Stage I | T2 | MX | N0 |
| TCGA-05-4415 | 91 | 1 | 57 | MALE | unknow | Stage IIIB | T4 | M0 | N2 |
| TCGA-86-A4JF | 737 | 1 | 56 | MALE | unknow | Stage IIB | T3 | M0 | N0 |
| TCGA-86-7701 | 947 | 0 | 66 | MALE | unknow | Stage IV | T2 | M1 | N0 |
| TCGA-44-3918 | 1036 | 0 | 60 | FEMALE | unknow | Stage IA | T1 | M0 | N0 |
| TCGA-50-6597 | 1268 | 1 | 79 | FEMALE | unknow | Stage IB | T2 | M0 | N0 |
| TCGA-NJ-A4YI | 4 | 1 | 87 | FEMALE | unknow | Stage IIIA | T2 | M0 | N2 |
| TCGA-86-8280 | 701 | 0 | 54 | FEMALE | unknow | Stage IIA | T2b | M0 | N0 |
| TCGA-44-2666 | 97 | 1 | 43 | MALE | unknow | Stage IB | T2 | M0 | N0 |
| TCGA-78-7154 | 593 | 1 | 72 | MALE | unknow | Stage IIIA | T3 | M0 | N2 |
| TCGA-55-A491 | 626 | 0 | 81 | FEMALE | unknow | Stage IA | T1b | MX | N0 |
| TCGA-05-4390 | 1126 | 0 | 58 | FEMALE | unknow | Stage IB | T2 | M0 | N0 |
| TCGA-05-4382 | 607 | 0 | 68 | MALE | unknow | Stage IB | T2 | M0 | N0 |
| TCGA-55-8206 | 888 | 0 | 56 | MALE | unknow | Stage IA | T1b | M0 | N0 |
| TCGA-55-7576 | 670 | 0 | 54 | MALE | unknow | Stage IB | T2a | M0 | N0 |
| TCGA-55-8506 | 11 | 0 | 62 | FEMALE | unknow | Stage IIB | T3 | MX | N0 |
| TCGA-50-5930 | 282 | 1 | 47 | MALE | unknow | Stage IIIA | T2 | M0 | N2 |
| TCGA-05-4403 | 578 | 0 | 76 | MALE | unknow | Stage IB | T2 | M0 | N0 |
| TCGA-55-7911 | 537 | 0 | 70 | FEMALE | unknow | Stage IA | T1a | MX | N0 |
| TCGA-78-7143 | 4961 | 1 | 62 | FEMALE | unknow | Stage IB | T2 | M0 | N0 |
| TCGA-62-A46P | 594 | 1 | 65 | MALE | unknow | Stage IB | T2 | M0 | N0 |
| TCGA-95-8494 | 84 | 0 | 67 | MALE | unknow | Stage IIA | T2a | M0 | N1 |
| TCGA-35-5375 | 264 | 0 | 61 | MALE | unknow | Stage IIIA | T2 | M0 | N2 |
| TCGA-78-7163 | 7248 | 0 | 60 | MALE | unknow | Stage IB | T2 | M0 | N0 |
| TCGA-05-4417 | 455 | 0 | 51 | FEMALE | unknow | Stage IB | T2 | M0 | N0 |
| TCGA-91-6828 | 323 | 0 | 70 | MALE | unknow | Stage IA | T1a | M0 | N0 |
| TCGA-44-A47A | 466 | 0 | 78 | FEMALE | unknow | Stage IB | T2a | MX | N0 |
| TCGA-L9-A443 | 193 | 1 | 63 | FEMALE | unknow | Stage IA | T1a | MX | N0 |
| TCGA-86-A4D0 | 116 | 1 | 48 | MALE | unknow | Stage IIA | T2b | M0 | N0 |
| TCGA-44-A479 | 486 | 0 | 73 | FEMALE | unknow | Stage IB | T2 | MX | N0 |
| TCGA-97-A4LX | 614 | 0 | 81 | MALE | unknow | Stage IB | T2a | M0 | N0 |
| TCGA-49-4486 | 2318 | 1 | 72 | MALE | unknow | Stage IA | T1 | M0 | N0 |
| TCGA-50-5932 | 1235 | 1 | 75 | MALE | unknow | Stage IIB | T2 | M0 | N1 |
| TCGA-86-8073 | 740 | 0 | 58 | MALE | unknow | Stage IB | T2a | M0 | N0 |
| TCGA-NJ-A55O | 13 | 0 | 56 | FEMALE | unknow | Stage IIA | T1b | M0 | N1 |
| TCGA-55-A494 | 481 | 0 | 61 | FEMALE | unknow | Stage IB | T2a | MX | N0 |
| TCGA-69-7765 | 165 | 0 | 56 | MALE | unknow | unknow | T4 | MX | N0 |
| TCGA-86-8672 | 19 | 1 | 59 | MALE | unknow | Stage IIB | T3 | M0 | N0 |
| TCGA-49-4488 | 869 | 1 | 74 | FEMALE | unknow | Stage IA | T1 | MX | N0 |
| TCGA-55-7903 | 567 | 0 | 64 | MALE | unknow | Stage IA | T1b | MX | N0 |
| TCGA-MP-A4T2 | 1136 | 1 | 71 | MALE | unknow | Stage IA | T1 | M0 | N0 |
| TCGA-49-4487 | 855 | 1 | 72 | FEMALE | unknow | Stage IA | T1 | M0 | N0 |
| TCGA-99-8033 | 656 | 1 | 74 | FEMALE | unknow | Stage IV | TX | M1 | NX |
| TCGA-50-5068 | 1499 | 1 | 59 | FEMALE | unknow | Stage IIB | T2 | MX | N1 |
| TCGA-55-6978 | 176 | 1 | 81 | MALE | unknow | Stage IIA | T2b | MX | N0 |
| TCGA-91-A4BC | 44 | 0 | 59 | MALE | unknow | Stage IIA | T2b | MX | N0 |
| TCGA-49-4490 | 385 | 1 | 45 | FEMALE | unknow | Stage IIIA | T3 | M0 | N2 |
| TCGA-86-7955 | 1072 | 0 | 62 | MALE | unknow | Stage IB | T2a | M0 | N0 |
| TCGA-05-4430 | 761 | 0 | 59 | FEMALE | unknow | Stage IB | T2 | M0 | N0 |
| TCGA-44-3398 | 1163 | 0 | 77 | FEMALE | unknow | Stage IA | T1b | M0 | N0 |
| TCGA-49-4507 | 268 | 1 | 73 | FEMALE | unknow | Stage IIIA | T3 | M0 | N1 |
| TCGA-97-A4M5 | 634 | 0 | 83 | MALE | unknow | Stage IA | T1b | M0 | N0 |
| TCGA-49-6745 | 522 | 0 | 82 | MALE | unknow | Stage IIIA | T2a | M0 | N2 |
| TCGA-86-8674 | 806 | 0 | 50 | MALE | unknow | Stage IIA | T2a | M0 | N1 |
| TCGA-05-4384 | 426 | 0 | 66 | MALE | unknow | Stage IIIA | T2 | M0 | N2 |
| TCGA-69-7979 | 408 | 0 | 71 | FEMALE | unknow | Stage IB | T2a | MX | N0 |
| TCGA-78-7147 | 586 | 1 | 67 | FEMALE | unknow | Stage IIB | T2 | M0 | N1 |
| TCGA-93-7347 | 683 | 0 | 76 | FEMALE | unknow | Stage IA | T1a | MX | N0 |
| TCGA-49-4514 | 1700 | 0 | 79 | FEMALE | unknow | Stage IA | T1 | M0 | N0 |
| TCGA-49-6742 | 488 | 1 | 70 | MALE | unknow | Stage IIA | T2a | M0 | N1 |
| TCGA-MN-A4N1 | 827 | 0 | 60 | MALE | unknow | Stage IIA | T2a | M0 | N1 |
| TCGA-J2-A4AD | 550 | 1 | 61 | FEMALE | unknow | Stage IA | T1a | MX | N0 |
| TCGA-78-7160 | 697 | 1 | 61 | MALE | unknow | Stage IV | T4 | M1 | N2 |
| TCGA-05-4397 | 731 | 1 | 65 | MALE | unknow | Stage IIB | T2 | M0 | N1 |
| TCGA-91-8497 | 434 | 1 | 75 | FEMALE | unknow | Stage IA | T1a | MX | N0 |
| TCGA-55-7283 | 609 | 0 | 76 | FEMALE | unknow | Stage IIIA | T3 | MX | N2 |
| TCGA-05-4244 | 0 | 0 | 70 | MALE | unknow | Stage IV | T2 | M1 | N2 |
| TCGA-91-6848 | 224 | 0 | 59 | MALE | unknow | Stage IIIA | T2 | MX | N2 |
| TCGA-MP-A4TD | 307 | 1 | 71 | MALE | unknow | Stage IIIA | T2 | M0 | N2 |
| TCGA-55-8620 | 375 | 1 | 60 | MALE | unknow | Stage IV | T1a | M1b | N1 |
| TCGA-97-7547 | 1965 | 0 | 67 | FEMALE | unknow | Stage IB | T2 | MX | N0 |
| TCGA-44-6777 | 987 | 1 | 85 | FEMALE | unknow | Stage IB | T2 | MX | NX |
| TCGA-38-4629 | 864 | 1 | 68 | MALE | unknow | Stage IIB | T3 | M0 | N0 |
| TCGA-55-8097 | 476 | 0 | 60 | FEMALE | unknow | Stage IA | T1a | MX | N0 |
| TCGA-97-8179 | 435 | 0 | 72 | MALE | unknow | Stage IA | T1a | M0 | N0 |
| TCGA-78-8648 | 1209 | 1 | 58 | FEMALE | unknow | Stage IIB | T3 | M0 | N0 |
| TCGA-75-5125 | 2027 | 1 | unknow | MALE | unknow | Stage IIB | T2 | M0 | N1 |
| TCGA-95-7944 | 377 | 0 | 71 | MALE | unknow | Stage IA | T1a | M0 | N0 |
| TCGA-44-6147 | 845 | 0 | 67 | FEMALE | unknow | Stage IA | T1b | M0 | NX |
| TCGA-55-7727 | 119 | 0 | 70 | MALE | unknow | Stage IIIA | T1a | MX | N2 |
| TCGA-95-7039 | 1272 | 0 | 54 | FEMALE | unknow | Stage IIB | T3 | MX | N0 |
| TCGA-55-6975 | 118 | 1 | 61 | MALE | unknow | Stage IIB | T2 | M0 | N1 |
| TCGA-05-4433 | 730 | 0 | 82 | MALE | unknow | Stage IB | T2 | M0 | N0 |
| TCGA-05-4427 | 791 | 0 | 65 | FEMALE | unknow | Stage IIB | T2 | M0 | N1 |
| TCGA-86-7711 | 1046 | 1 | 70 | MALE | unknow | Stage IIA | T2a | M0 | N1 |
| TCGA-69-7761 | 186 | 0 | 84 | MALE | unknow | Stage IB | T2a | MX | N0 |
| TCGA-44-2661 | 1159 | 0 | 69 | FEMALE | unknow | Stage IA | T1 | M0 | N0 |
| TCGA-55-6981 | 1379 | 1 | 53 | FEMALE | unknow | Stage IIIA | T1 | M0 | N2 |
| TCGA-MP-A4TH | 741 | 0 | 70 | FEMALE | unknow | Stage IA | T1a | M0 | N0 |
| TCGA-55-8299 | 469 | 1 | 61 | FEMALE | unknow | Stage IA | T1b | MX | N0 |
| TCGA-L4-A4E6 | 435 | 0 | 67 | MALE | unknow | Stage IA | T1 | M0 | N0 |
| TCGA-44-A47F | 337 | 0 | 74 | MALE | unknow | Stage IIB | T2b | M0 | N1 |
| TCGA-55-7913 | 561 | 1 | 61 | FEMALE | unknow | Stage IA | T1b | MX | N0 |
| TCGA-38-4628 | 1492 | 1 | 65 | FEMALE | unknow | Stage IIB | T2 | M0 | N1 |
| TCGA-67-6215 | 174 | 0 | 52 | FEMALE | unknow | Stage IB | T2a | M0 | N0 |
| TCGA-NJ-A7XG | 617 | 0 | 49 | MALE | unknow | Stage IIIA | T4 | M0 | N1 |
| TCGA-67-3772 | 573 | 0 | 82 | FEMALE | unknow | Stage IB | T2 | M0 | N0 |
| TCGA-S2-AA1A | 513 | 0 | 68 | FEMALE | unknow | Stage IA | T1b | M0 | N0 |
| TCGA-49-6767 | 677 | 0 | 46 | FEMALE | unknow | Stage IIB | T3 | MX | N0 |
| TCGA-55-6980 | 2109 | 0 | 56 | MALE | unknow | Stage IA | T1 | M0 | N0 |
| TCGA-44-6144 | 723 | 0 | 58 | MALE | unknow | Stage IA | T1a | M0 | N0 |
| TCGA-44-5645 | 852 | 0 | 61 | FEMALE | unknow | Stage IA | T1 | unknow | NX |
| TCGA-MP-A4T7 | 167 | 1 | 75 | FEMALE | unknow | Stage IV | T2 | M1 | N0 |
| TCGA-62-A46V | 2199 | 0 | 78 | FEMALE | unknow | Stage IB | T2 | M0 | N0 |
| TCGA-44-6774 | 658 | 0 | 56 | FEMALE | unknow | Stage IIIA | T1 | M0 | N2 |
| TCGA-55-8621 | 515 | 0 | 75 | FEMALE | unknow | Stage IA | T1a | MX | N0 |
| TCGA-05-4396 | 303 | 1 | 76 | MALE | unknow | Stage IIIB | T4 | M0 | N1 |
| TCGA-55-7573 | 487 | 0 | 72 | FEMALE | unknow | Stage IA | T1b | MX | N0 |
| TCGA-49-AARR | 4992 | 0 | 68 | MALE | unknow | Stage IA | T1 | MX | N0 |
| TCGA-97-A4M3 | 540 | 0 | 69 | FEMALE | unknow | Stage IA | T1b | M0 | N0 |
| TCGA-49-AARO | 3759 | 0 | 39 | FEMALE | unknow | Stage IA | T1a | MX | N0 |
| TCGA-91-6830 | 60 | 0 | 65 | FEMALE | unknow | Stage IIA | T1 | MX | N1 |
| TCGA-38-6178 | 448 | 0 | 70 | FEMALE | unknow | Stage IIIA | T2b | unknow | N2 |
| TCGA-55-8091 | 600 | 0 | 74 | MALE | unknow | Stage IB | T2 | MX | N0 |
| TCGA-55-8507 | 418 | 0 | 53 | MALE | unknow | Stage IA | T1a | MX | N0 |
| TCGA-75-7030 |  | 0 | unknow | MALE | unknow | Stage IIB | T3 | M0 | N0 |
| TCGA-86-A456 | 896 | 0 | 78 | FEMALE | unknow | Stage IA | T1a | M0 | N0 |
| TCGA-55-A492 | 596 | 0 | 70 | FEMALE | unknow | Stage IA | T1a | MX | N0 |
| TCGA-64-1678 | 1189 | 0 | 70 | FEMALE | unknow | unknow | T2b | M0 | N0 |
| TCGA-62-8399 | 2696 | 0 | 62 | MALE | unknow | Stage IIIA | T2 | M0 | N2 |
| TCGA-55-6985 | 1233 | 0 | 58 | FEMALE | unknow | Stage IB | T2 | MX | N0 |
| TCGA-44-8120 | 260 | 0 | 58 | MALE | unknow | Stage IB | T2a | M0 | N0 |
| TCGA-50-5935 | 653 | 1 | 86 | FEMALE | unknow | Stage IA | T1 | M0 | N0 |
| TCGA-50-5044 | 624 | 1 | 72 | FEMALE | unknow | Stage IIIB | T4 | M0 | N1 |
| TCGA-55-8085 | 904 | 0 | 64 | MALE | unknow | Stage IA | T1b | M0 | N0 |
| TCGA-L9-A444 | 307 | 0 | 60 | FEMALE | unknow | Stage IA | T1a | MX | N0 |
| TCGA-55-8615 | 446 | 0 | 67 | MALE | unknow | Stage IIIA | T3 | MX | N2 |
| TCGA-97-A4M0 | 652 | 0 | 60 | FEMALE | unknow | Stage IB | T2a | M0 | N0 |
| TCGA-95-7562 | 87 | 1 | 71 | MALE | unknow | Stage IIA | T2a | M0 | N1 |
| TCGA-38-4626 | 3674 | 0 | 57 | FEMALE | unknow | unknow | T2b | M0 | N0 |
| TCGA-67-6216 | 141 | 0 | 57 | FEMALE | unknow | Stage IA | T1a | M0 | N0 |
| TCGA-75-6203 |  | 0 | unknow | FEMALE | unknow | Stage IIIA | T2 | M0 | N2 |
| TCGA-67-3773 | 427 | 0 | 84 | FEMALE | unknow | Stage IB | T2 | M0 | N0 |
| TCGA-78-7161 | 291 | 1 | 69 | FEMALE | unknow | Stage IIB | T3 | M0 | N0 |
| TCGA-NJ-A4YQ | 1432 | 0 | 69 | FEMALE | unknow | Stage IA | T1b | M0 | N0 |
| TCGA-50-5072 | 250 | 1 | 74 | MALE | unknow | Stage IIIA | T2 | M0 | N2 |
| TCGA-50-5931 | 434 | 1 | 75 | FEMALE | unknow | Stage IB | T2 | M0 | N0 |
| TCGA-49-AARN | 1135 | 1 | 56 | FEMALE | unknow | Stage IA | T1 | MX | N0 |
| TCGA-64-1681 | 1167 | 1 | 61 | FEMALE | unknow | Stage IA | T1 | M0 | N0 |
| TCGA-99-7458 | 747 | 0 | 74 | FEMALE | unknow | Stage IIIA | T4 | M0 | N0 |
| TCGA-35-4122 | 225 | 0 | 69 | MALE | unknow | Stage IA | T1 | M0 | N0 |
| TCGA-55-7728 | 704 | 0 | 64 | FEMALE | unknow | Stage IB | T2a | MX | N0 |
| TCGA-44-7672 | 719 | 0 | 52 | FEMALE | unknow | Stage IA | T1b | M0 | N0 |
| TCGA-44-7662 | 218 | 0 | 61 | MALE | unknow | Stage IB | T2a | MX | N0 |
| TCGA-86-7714 | 625 | 1 | 61 | FEMALE | unknow | Stage IIIA | T1b | M0 | N2 |
| TCGA-97-7552 | 1932 | 0 | 70 | MALE | unknow | Stage IB | T2 | MX | N0 |
| TCGA-44-5643 | 1013 | 0 | 53 | MALE | unknow | Stage IIIA | T2b | M0 | N2 |
| TCGA-75-7025 | 3305 | 0 | unknow | MALE | unknow | Stage IB | T2 | M0 | N0 |
| TCGA-78-8655 | 2360 | 0 | 77 | FEMALE | unknow | Stage IA | T1 | M0 | N0 |
| TCGA-97-7937 | 564 | 0 | 65 | MALE | unknow | Stage IB | T2a | MX | N0 |
| TCGA-64-1679 | 2488 | 0 | 58 | FEMALE | unknow | Stage IIIA | T1 | M0 | N2 |
| TCGA-86-A4P8 | 805 | 0 | 59 | FEMALE | unknow | Stage IIIA | T1b | MX | N2 |
| TCGA-55-8205 | 599 | 0 | 76 | FEMALE | unknow | Stage IIA | T2b | M0 | N0 |
| TCGA-44-2668 | 761 | 1 | 51 | MALE | unknow | Stage IB | T2 | M0 | N0 |
| TCGA-NJ-A4YP | 50 | 0 | 52 | MALE | unknow | Stage IB | T2a | M0 | N0 |
| TCGA-49-4494 | 1081 | 1 | 77 | MALE | unknow | Stage IIIA | T3 | M0 | N2 |
| TCGA-49-AAR9 | 260 | 1 | 61 | MALE | unknow | Stage IIB | T3 | MX | N0 |
| TCGA-38-4625 | 2973 | 0 | 66 | FEMALE | unknow | Stage IB | T2a | M0 | N0 |
| TCGA-44-7661 | 557 | 1 | 69 | FEMALE | unknow | Stage IB | T2a | M0 | N0 |
| TCGA-55-7726 | 652 | 0 | 72 | FEMALE | unknow | Stage IA | T1b | MX | N0 |
| TCGA-75-6205 |  | 1 | unknow | FEMALE | unknow | Stage IB | T2a | M0 | N0 |
| TCGA-55-6972 | 1632 | 1 | 72 | MALE | unknow | Stage IB | T2 | M0 | N0 |
| TCGA-86-8359 | 444 | 1 | 52 | MALE | unknow | Stage IIIA | T3 | M0 | N2 |
| TCGA-50-6673 | 22 | 1 | 84 | FEMALE | unknow | Stage I | T1 | M0 | N0 |
| TCGA-44-3917 | 1183 | 0 | 33 | FEMALE | unknow | Stage IB | T2 | M0 | N0 |
| TCGA-95-7567 | 568 | 0 | 61 | MALE | unknow | Stage IIB | T2b | M0 | N1 |
| TCGA-97-8176 | 468 | 1 | 63 | MALE | unknow | Stage IIIA | T3 | M0 | N1 |
| TCGA-64-1677 | 628 | 1 | 77 | FEMALE | unknow | Stage IIIA | T2 | M0 | N2 |
| TCGA-50-5942 | 1847 | 0 | 67 | FEMALE | unknow | Stage IA | T1 | M0 | N0 |
| TCGA-38-4627 | 1147 | 1 | 64 | FEMALE | unknow | Stage IIA | T1b | M0 | N1 |
| TCGA-86-8055 | 124 | 1 | 79 | MALE | unknow | Stage IIA | T2a | M0 | N1 |
| TCGA-55-A4DF | 440 | 1 | 88 | MALE | unknow | Stage IA | T1b | MX | N0 |
| TCGA-44-2664 | 1251 | 0 | 66 | FEMALE | unknow | Stage IA | T1 | M0 | N0 |
| TCGA-44-A47B | 287 | 0 | 79 | MALE | unknow | Stage IB | T2a | M0 | N0 |
| TCGA-62-A46R | 1725 | 1 | 54 | FEMALE | unknow | Stage IB | T2 | M0 | N0 |
| TCGA-71-8520 | 210 | 1 | 60 | FEMALE | unknow | Stage IB | T2 | M0 | N0 |
| TCGA-80-5611 | 2595 | 0 | unknow | MALE | unknow | Stage IB | T2 | M0 | N0 |
| TCGA-05-4250 | 121 | 1 | 79 | FEMALE | unknow | Stage IIIA | T3 | M0 | N1 |
| TCGA-78-7158 | 179 | 1 | 59 | FEMALE | unknow | Stage IIIB | T4 | M0 | N2 |
| TCGA-53-7813 | 424 | 0 | 51 | FEMALE | unknow | Stage IIIB | T4 | M0 | N0 |
| TCGA-55-6970 | 464 | 1 | 67 | FEMALE | unknow | Stage IIIA | T2 | MX | N2 |
| TCGA-73-A9RS | 340 | 1 | 41 | MALE | unknow | Stage IIB | T3 | M0 | N0 |
| TCGA-69-7764 | 414 | 0 | 75 | MALE | unknow | Stage IA | T1b | M0 | N0 |
| TCGA-55-8094 | 541 | 0 | 51 | MALE | unknow | Stage IV | T2b | M1b | N0 |
| TCGA-95-A4VN | 553 | 0 | 62 | FEMALE | unknow | Stage IIA | T2a | M0 | N1 |
| TCGA-69-8254 | 409 | 0 | 85 | MALE | unknow | unknow | T2b | unknow | unknow |
| TCGA-49-AAR3 | 1893 | 0 | 69 | MALE | unknow | Stage IIB | T2 | MX | N1 |
| TCGA-49-4510 | 896 | 1 | 51 | FEMALE | unknow | Stage IIB | T2 | M0 | N1 |
| TCGA-55-7281 | 872 | 0 | 70 | FEMALE | unknow | Stage IA | T1b | M0 | N0 |
| TCGA-69-7763 | 690 | 0 | 69 | MALE | unknow | Stage IA | T1b | M0 | N0 |
| TCGA-78-7148 | 626 | 1 | 71 | MALE | unknow | Stage IIB | T2 | M0 | N1 |
| TCGA-97-A4M7 | 629 | 0 | 74 | MALE | unknow | Stage IA | T1b | M0 | N0 |
| TCGA-86-8668 | 423 | 0 | 61 | FEMALE | unknow | Stage IA | T1b | M0 | N0 |
| TCGA-49-AAQV | 677 | 1 | 63 | FEMALE | unknow | Stage II | T1 | MX | N1 |
| TCGA-71-6725 | 256 | 0 | 48 | FEMALE | unknow | Stage IB | T2 | M0 | N0 |
| TCGA-55-7570 | 824 | 0 | 60 | MALE | unknow | Stage IA | T1a | MX | N0 |
| TCGA-05-4402 | 244 | 1 | 57 | FEMALE | unknow | Stage IV | T2 | M1 | NX |
| TCGA-67-4679 | 448 | 0 | 69 | MALE | unknow | unknow | T3 | M0 | N0 |
| TCGA-05-4422 | 365 | 0 | 68 | MALE | unknow | Stage IB | T2 | M0 | N0 |
| TCGA-55-7227 | 952 | 1 | 77 | MALE | unknow | Stage IIIA | T3 | MX | N1 |
| TCGA-55-1594 | 1178 | 0 | 68 | MALE | unknow | Stage IIIA | T2 | M0 | N2 |
| TCGA-49-AAR4 | 879 | 1 | 51 | MALE | unknow | Stage IIIA | T2 | MX | N2 |
| TCGA-62-A46Y | 414 | 1 | 70 | FEMALE | unknow | Stage IIIA | T2 | M0 | N2 |
| TCGA-86-8054 | 1148 | 0 | 61 | MALE | unknow | Stage IIB | T2b | M0 | N1 |
| TCGA-62-A46S | 1653 | 1 | 73 | MALE | unknow | Stage IB | T2 | M0 | N0 |
| TCGA-97-8177 | 499 | 0 | 59 | FEMALE | unknow | Stage IB | T2a | M0 | N0 |
| TCGA-05-5425 | 882 | 0 | 68 | MALE | unknow | Stage IIB | T2b | M0 | N1 |
| TCGA-49-6744 | 1683 | 0 | 64 | FEMALE | unknow | Stage IIA | T2a | MX | N1 |
| TCGA-44-6778 | 1864 | 0 | 59 | MALE | unknow | Stage IA | T1 | MX | N0 |
| TCGA-75-6206 | 2590 | 0 | unknow | MALE | unknow | Stage IB | T2 | M0 | N0 |
| TCGA-80-5607 |  | 0 | unknow | FEMALE | unknow | Stage IIB | T2 | M0 | N1 |
| TCGA-NJ-A4YF | 2161 | 0 | 50 | FEMALE | unknow | Stage IA | T1 | M0 | N0 |
| TCGA-78-7220 | 807 | 1 | 53 | FEMALE | unknow | Stage IIIA | T2 | M0 | N2 |
| TCGA-50-6595 | 189 | 1 | 74 | FEMALE | unknow | Stage IIIA | T2 | M0 | N2 |
| TCGA-78-7167 | 2681 | 1 | 77 | MALE | unknow | Stage IV | T2 | M1 | N0 |
| TCGA-95-7948 | 476 | 0 | 42 | FEMALE | unknow | Stage IB | T2a | M0 | N0 |
| TCGA-75-6207 |  | 1 | unknow | MALE | unknow | Stage IIIA | T2 | M0 | N2 |
| TCGA-05-4389 | 1369 | 0 | 70 | MALE | unknow | Stage IA | T1 | M0 | N0 |
| TCGA-05-5429 | 275 | 1 | 60 | MALE | unknow | Stage IIIA | T3 | M0 | N2 |
| TCGA-55-7907 | 343 | 1 | 77 | MALE | unknow | Stage IIA | T2a | MX | N1 |
| TCGA-67-3776 | 61 | 0 | 57 | FEMALE | unknow | Stage IIB | T2 | M0 | N1 |
| TCGA-73-4677 | 38 | 1 | 74 | MALE | unknow | unknow | T2a | M0 | N0 |
| TCGA-97-8172 | 545 | 0 | 75 | FEMALE | unknow | Stage IB | T2a | M0 | N0 |
| TCGA-75-5147 | 1333 | 0 | unknow | FEMALE | unknow | Stage IB | T2 | M0 | N0 |
| TCGA-50-5045 | 2174 | 1 | 57 | FEMALE | unknow | unknow | T2 | M0 | N1 |
| TCGA-44-A4SU | 409 | 1 | 67 | FEMALE | unknow | Stage IA | T1a | MX | N0 |
| TCGA-55-8301 | 534 | 0 | 58 | MALE | unknow | Stage IB | T2a | MX | N0 |
| TCGA-55-1596 | 2065 | 0 | 55 | MALE | unknow | Stage IIB | T2 | M0 | N1 |
| TCGA-55-A4DG | 608 | 0 | 71 | MALE | unknow | Stage IA | T1b | MX | N0 |
| TCGA-49-AARE | 1229 | 1 | 51 | FEMALE | unknow | Stage IA | T1 | MX | N0 |
| TCGA-50-6592 | 777 | 1 | 71 | FEMALE | unknow | Stage IB | T2 | M0 | N0 |
| TCGA-J2-A4AG | 988 | 0 | 66 | FEMALE | unknow | Stage IA | T1b | MX | N0 |
| TCGA-67-3771 | 610 | 0 | 77 | FEMALE | unknow | Stage IA | T1 | M0 | N0 |
| TCGA-73-4662 | 2515 | 0 | 65 | FEMALE | unknow | Stage IA | T1 | M0 | N0 |
| TCGA-05-5420 | 457 | 0 | 67 | MALE | unknow | Stage IIIA | T2 | M0 | N2 |
| TCGA-86-8056 | 139 | 0 | 63 | FEMALE | unknow | Stage IIIA | T4 | M0 | N0 |
| TCGA-J2-A4AE | 1079 | 0 | 77 | FEMALE | unknow | Stage IA | T1a | MX | N0 |
| TCGA-44-7669 | 574 | 1 | 59 | MALE | unknow | Stage IIA | T1b | MX | N1 |
| TCGA-44-8119 | 285 | 0 | 73 | MALE | unknow | Stage IIB | T3 | M0 | N0 |
| TCGA-50-5941 | 1474 | 0 | 55 | FEMALE | unknow | Stage IIIA | T2a | M0 | N2 |
| TCGA-64-5781 | 1559 | 0 | 55 | FEMALE | unknow | Stage IB | T2 | M0 | N0 |
| TCGA-73-4666 | 800 | 0 | 52 | FEMALE | unknow | Stage IV | T1 | M1 | N0 |
| TCGA-L9-A8F4 | 476 | 0 | 64 | FEMALE | unknow | Stage IB | T2a | MX | N0 |
| TCGA-69-8253 | 426 | 0 | 59 | FEMALE | unknow | Stage IIA | T1a | MX | N1 |
| TCGA-97-8552 | 626 | 0 | 55 | FEMALE | unknow | Stage I | T1a | MX | N0 |
| TCGA-44-6776 | 2616 | 0 | 60 | FEMALE | unknow | Stage IA | T1 | MX | N0 |
| TCGA-L9-A5IP | 58 | 1 | 40 | FEMALE | unknow | Stage IV | T3 | M1b | N2 |
| TCGA-44-6148 | 704 | 0 | 60 | MALE | unknow | Stage IA | T1b | M0 | N0 |
| TCGA-38-4630 | 1073 | 1 | 75 | FEMALE | unknow | Stage IB | T2 | M0 | N0 |
| TCGA-95-7947 | 477 | 0 | 67 | MALE | unknow | Stage IA | T1a | M0 | N0 |
| TCGA-4B-A93V | 300 | 1 | 52 | FEMALE | unknow | Stage IA | T1b | M0 | N0 |
| TCGA-86-8076 | 993 | 0 | 42 | MALE | unknow | Stage IA | T1 | M0 | N0 |
| TCGA-05-4410 | 0 | 0 | 62 | MALE | unknow | Stage IB | T2 | M0 | N0 |
| TCGA-62-A472 | 910 | 0 | 70 | MALE | unknow | Stage IIB | T3 | M0 | N0 |
| TCGA-55-6968 | 1293 | 1 | 61 | MALE | unknow | Stage IV | T1 | M1 | N0 |
| TCGA-55-8514 | 520 | 0 | 70 | FEMALE | unknow | Stage IB | T2a | MX | N0 |
| TCGA-75-7031 |  | 0 | unknow | FEMALE | unknow | Stage IB | T2 | M0 | N0 |
| TCGA-62-8397 | 1289 | 0 | 70 | FEMALE | unknow | Stage IIB | T3 | M0 | N0 |
| TCGA-86-7953 | 997 | 0 | 69 | FEMALE | unknow | Stage IA | T1b | M0 | N0 |
| TCGA-75-5146 | 2368 | 0 | unknow | MALE | unknow | Stage IB | T2 | M0 | N0 |
| TCGA-44-A47G | 351 | 0 | 73 | FEMALE | unknow | Stage IA | T1 | M0 | N0 |
| TCGA-86-8671 | 839 | 0 | 72 | FEMALE | unknow | Stage IIB | T2b | M0 | N1 |
| TCGA-86-8358 | 653 | 0 | 44 | MALE | unknow | Stage IB | T2a | M0 | N0 |
| TCGA-93-A4JN | 718 | 0 | 71 | MALE | unknow | Stage IV | T2a | M1a | N0 |
| TCGA-05-4432 | 761 | 0 | 66 | MALE | unknow | Stage IIB | T2 | M0 | N1 |
| TCGA-86-8669 | 938 | 0 | 64 | MALE | unknow | Stage IA | T1b | M0 | N0 |
| TCGA-95-A4VK | 651 | 0 | 74 | FEMALE | unknow | Stage IIIA | T2b | M0 | N2 |
| TCGA-44-7670 | 882 | 0 | 47 | FEMALE | unknow | Stage IIA | T1b | M0 | N1 |
| TCGA-73-7499 | 1531 | 1 | 81 | FEMALE | unknow | Stage IB | T2a | M0 | N0 |
| TCGA-62-A46U | 2067 | 0 | 71 | FEMALE | unknow | Stage IIB | T2 | M0 | N1 |
| TCGA-55-8508 | 617 | 0 | 60 | FEMALE | unknow | Stage IIA | T2a | MX | N1 |
| TCGA-44-2657 | 1351 | 0 | 74 | FEMALE | unknow | Stage IB | T2 | M0 | NX |
| TCGA-97-8171 | 568 | 0 | 81 | MALE | unknow | Stage IV | T2a | M1a | N2 |
| TCGA-50-5944 | 1750 | 0 | 69 | FEMALE | unknow | Stage IA | T1 | M0 | N0 |
| TCGA-78-7633 | 1528 | 1 | 67 | MALE | unknow | Stage IB | T2 | M0 | N0 |
| TCGA-64-5775 | 62 | 1 | 71 | MALE | unknow | Stage IIIA | T4 | M0 | N0 |
| TCGA-J2-8194 | 724 | 0 | 69 | FEMALE | unknow | Stage IIB | T3 | MX | N0 |
| TCGA-78-7539 | 791 | 0 | 75 | FEMALE | unknow | Stage IIA | T2b | M0 | N0 |
| TCGA-91-6829 | 1258 | 1 | 78 | MALE | unknow | Stage IB | T2 | MX | N0 |
| TCGA-99-8028 | 1118 | 0 | 50 | FEMALE | unknow | Stage IA | T1a | M0 | N0 |
| TCGA-78-7153 | 3635 | 0 | 65 | FEMALE | unknow | Stage IB | T2 | M0 | N0 |
| TCGA-L9-A50W | 442 | 1 | 75 | MALE | unknow | Stage IIA | T1b | MX | N1 |
| TCGA-50-6594 | 370 | 1 | 79 | FEMALE | unknow | Stage IIIA | T3 | M0 | N2 |
| TCGA-50-5936 | 257 | 1 | 58 | MALE | unknow | Stage IIIA | T2 | M0 | N2 |
| TCGA-05-5428 | 670 | 0 | 57 | MALE | unknow | Stage IIA | T1b | M0 | N1 |
| TCGA-55-5899 | 930 | 0 | 58 | MALE | unknow | unknow | T1a | M0 | N1 |
| TCGA-44-7659 | 691 | 0 | 70 | MALE | unknow | Stage IA | T1b | MX | N0 |
| TCGA-55-7816 | 468 | 1 | 49 | FEMALE | unknow | Stage IV | TX | MX | NX |
| TCGA-05-4425 | 669 | 0 | 70 | FEMALE | unknow | Stage IV | T2 | M1 | N0 |
| TCGA-44-2665 | 1301 | 0 | 55 | FEMALE | unknow | Stage IIB | T2 | M0 | N1 |
| TCGA-05-4424 | 913 | 0 | 70 | MALE | unknow | Stage IIB | T3 | M0 | N0 |
| TCGA-78-7156 | 976 | 1 | 62 | MALE | unknow | Stage IV | T4 | M1 | N1 |
| TCGA-49-4512 | 905 | 1 | 69 | FEMALE | unknow | Stage IIIA | T2 | MX | N2 |
| TCGA-78-7536 | 244 | 1 | 69 | MALE | unknow | Stage IIIA | T2 | M0 | N2 |
| TCGA-97-8175 | 551 | 0 | 55 | FEMALE | unknow | Stage IB | T2a | M0 | N0 |
| TCGA-49-4501 | 1421 | 1 | 67 | FEMALE | unknow | Stage IB | T2 | M0 | N0 |
| TCGA-53-7626 | 929 | 1 | 76 | FEMALE | unknow | Stage IIA | T1 | M0 | N1 |
| TCGA-55-6984 | 760 | 1 | 71 | FEMALE | unknow | Stage IIB | T2 | M0 | N1 |
| TCGA-44-2659 | 1367 | 0 | 65 | FEMALE | unknow | Stage IIB | T1 | M0 | N1 |
| TCGA-MP-A4SW | 1778 | 1 | 53 | MALE | unknow | Stage IIB | T2 | M0 | N1 |
| TCGA-55-A48X | 689 | 0 | 63 | FEMALE | unknow | Stage IIA | T1b | M0 | N1 |
| TCGA-55-1592 | 701 | 1 | 65 | MALE | unknow | Stage IA | T2 | M0 | N0 |
| TCGA-86-8673 | 862 | 0 | 61 | MALE | unknow | Stage IB | T2 | M0 | N0 |
| TCGA-35-4123 | 182 | 0 | 38 | MALE | unknow | Stage IA | T1 | M0 | N0 |
| TCGA-78-8640 | 7062 | 0 | 59 | MALE | unknow | Stage IIA | T1 | M0 | N1 |
| TCGA-75-7027 | 3059 | 0 | unknow | MALE | unknow | Stage IB | T2 | M0 | N0 |
| TCGA-99-8032 | 44 | 0 | 61 | MALE | unknow | Stage IA | T1a | M0 | N0 |
| TCGA-49-6761 | 354 | 0 | 68 | FEMALE | unknow | Stage IIIA | T1 | MX | N2 |
| TCGA-MP-A4SY | 1501 | 1 | 61 | MALE | unknow | Stage IIB | T2 | M0 | N1 |
| TCGA-55-A490 | 99 | 1 | 78 | MALE | unknow | Stage IIA | T2b | MX | N0 |
| TCGA-93-7348 | 531 | 0 | 75 | FEMALE | unknow | Stage IA | T1a | MX | N0 |
| TCGA-49-AAR0 | 4765 | 0 | 57 | MALE | unknow | Stage IA | T1 | MX | N0 |
| TCGA-93-A4JP | 578 | 0 | 64 | MALE | unknow | Stage IV | TX | M1b | NX |
| TCGA-55-6979 | 237 | 1 | 59 | FEMALE | unknow | Stage IIB | T2 | M0 | N1 |
| TCGA-73-7498 | 1189 | 0 | 58 | FEMALE | unknow | Stage IA | T1b | M0 | N0 |
| TCGA-MP-A4T8 | 161 | 1 | 68 | MALE | unknow | Stage IIIA | T2 | M0 | N2 |
| TCGA-50-6590 | 1288 | 1 | 72 | FEMALE | unknow | Stage IB | T2 | M0 | N0 |
| TCGA-64-5815 | 866 | 0 | 74 | MALE | unknow | Stage IIB | T2 | M0 | N1 |
| TCGA-78-7540 | 1197 | 1 | 66 | FEMALE | unknow | Stage IB | T2 | M0 | N0 |
| TCGA-55-8510 | 539 | 0 | 55 | FEMALE | unknow | Stage IB | T2a | MX | N0 |
| TCGA-44-7671 | 889 | 0 | 64 | MALE | unknow | Stage IB | T2a | M0 | N0 |
| TCGA-91-8496 | 505 | 0 | 63 | FEMALE | unknow | Stage IB | T2a | MX | NX |
| TCGA-55-8089 | 702 | 1 | 56 | MALE | unknow | Stage IA | T1a | M0 | N0 |
| TCGA-99-AA5R | 658 | 0 | 70 | FEMALE | unknow | Stage IA | T1a | M0 | N0 |
| TCGA-55-8505 | 440 | 0 | 62 | MALE | unknow | Stage IIIA | T1a | MX | N2 |
| TCGA-05-5423 | 151 | 0 | 65 | MALE | unknow | Stage IIB | T2 | M0 | N1 |
| TCGA-55-8614 | 536 | 0 | 76 | MALE | unknow | Stage IB | T2a | MX | N0 |
| TCGA-50-5946 | 1617 | 0 | 62 | MALE | unknow | Stage IA | T1 | MX | N0 |
| TCGA-05-4245 | 730 | 0 | 81 | MALE | unknow | Stage IIIA | T2 | M0 | N2 |
| TCGA-91-A4BD | 603 | 0 | 78 | MALE | unknow | Stage IIA | T1b | MX | N1 |
| TCGA-55-8512 | 607 | 1 | 41 | MALE | unknow | Stage IV | T1a | M1b | N1 |
| TCGA-NJ-A55A | 15 | 0 | 76 | FEMALE | unknow | Stage IB | T2 | M0 | N0 |
| TCGA-05-4434 | 457 | 1 | 67 | FEMALE | unknow | Stage IV | T4 | M1 | N1 |
| TCGA-97-A4M6 | 568 | 0 | 45 | FEMALE | unknow | Stage IA | T1a | M0 | N0 |
| TCGA-86-7954 | 605 | 0 | 68 | FEMALE | unknow | Stage IB | T2 | M0 | N0 |
| TCGA-78-7150 | 666 | 1 | 59 | MALE | unknow | Stage IIB | T2 | M0 | N1 |
| TCGA-62-A470 | 1194 | 1 | 84 | MALE | unknow | Stage IB | T2 | M0 | N0 |
| TCGA-69-8255 | 129 | 0 | 71 | MALE | unknow | Stage IA | T1a | M0 | N0 |
| TCGA-55-8208 | 674 | 0 | 73 | FEMALE | unknow | Stage IA | T1b | M0 | N0 |
| TCGA-50-5051 | 478 | 1 | 42 | FEMALE | unknow | Stage IIIA | T2 | M0 | N2 |
| TCGA-55-7910 | 1040 | 0 | 50 | FEMALE | unknow | Stage IIA | T2b | M0 | N0 |
| TCGA-38-7271 | 800 | 1 | 72 | FEMALE | unknow | Stage IA | T1 | M0 | N0 |
| TCGA-86-8585 | 353 | 0 | 57 | MALE | unknow | Stage IB | T2a | M0 | N0 |
| TCGA-86-8075 | 694 | 1 | 66 | FEMALE | unknow | Stage IB | T2 | M0 | N0 |
| TCGA-73-4676 | 281 | 1 | 45 | MALE | unknow | Stage IIA | T2a | M0 | N1 |
| TCGA-50-5933 | 2393 | 1 | 72 | MALE | unknow | Stage IIIB | T4 | M0 | N2 |
| TCGA-73-4675 | 922 | 1 | 59 | MALE | unknow | Stage IIIA | T3 | M0 | N1 |
| TCGA-55-6987 | 2137 | 0 | 77 | MALE | unknow | Stage IA | T1 | M0 | N0 |
| TCGA-50-5055 | 1830 | 1 | 79 | FEMALE | unknow | Stage IIA | T1 | M0 | N1 |
| TCGA-MN-A4N5 | 84 | 0 | 63 | MALE | unknow | Stage IA | T1a | M0 | N0 |
| TCGA-38-A44F | 133 | 0 | 80 | MALE | unknow | Stage IB | T2a | M0 | N0 |
| TCGA-55-6971 | 1400 | 0 | 59 | FEMALE | unknow | Stage IB | T2 | MX | N0 |
| TCGA-69-7974 | 184 | 0 | 54 | FEMALE | unknow | Stage IIIA | T2a | MX | N2 |
| TCGA-44-6779 | 500 | 1 | 50 | FEMALE | unknow | Stage IIB | T2 | MX | N1 |
| TCGA-MP-A4TC | 74 | 1 | 77 | MALE | unknow | Stage IIIA | T1 | M0 | N2 |
| TCGA-55-8302 | 478 | 0 | 54 | MALE | unknow | Stage IB | T2 | MX | N0 |
| TCGA-67-3774 | 385 | 0 | 73 | FEMALE | unknow | Stage IB | T2 | M0 | N0 |
| TCGA-55-A493 | 28 | 0 | 54 | FEMALE | unknow | Stage IB | T2a | M0 | N0 |
| TCGA-50-7109 | 308 | 1 | 60 | MALE | unknow | Stage IA | T1 | M0 | N0 |
| TCGA-55-6982 | 995 | 1 | 79 | FEMALE | unknow | Stage IIB | T2 | M0 | N1 |
| TCGA-86-8074 | 24 | 0 | 62 | FEMALE | unknow | Stage IIA | T1b | M0 | N1 |
| TCGA-69-A59K | 591 | 0 | 60 | FEMALE | unknow | Stage IIB | T3 | M0 | N0 |
| TCGA-55-8090 | 598 | 1 | 80 | MALE | unknow | Stage IA | T1a | M0 | N0 |
| TCGA-97-A4M2 | 624 | 0 | 66 | MALE | unknow | Stage IA | T1a | M0 | N0 |
| TCGA-MP-A4T9 | 1265 | 1 | 54 | FEMALE | unknow | Stage IIIA | T2 | MX | N2 |
| TCGA-MP-A4TK | 582 | 1 | 56 | FEMALE | unknow | Stage IIB | T2 | MX | N1 |
| TCGA-91-6849 | 35 | 0 | 75 | FEMALE | unknow | Stage IIIA | T2 | MX | N2 |
| TCGA-50-6593 | 336 | 1 | 49 | FEMALE | unknow | Stage IIIA | T1 | M0 | N2 |
| TCGA-95-7043 | 503 | 1 | 63 | FEMALE | unknow | Stage IA | T1a | MX | N0 |
| TCGA-55-7724 | 705 | 0 | 76 | FEMALE | unknow | Stage IB | T2a | MX | N0 |
| TCGA-53-A4EZ | 1071 | 0 | 63 | MALE | unknow | Stage IIA | T2a | MX | N1 |
| TCGA-44-7667 | 1097 | 0 | 49 | FEMALE | unknow | Stage IIB | T3 | MX | N0 |
| TCGA-44-8117 | 385 | 0 | 54 | FEMALE | unknow | Stage IB | T2a | M0 | N0 |
| TCGA-55-6983 | 2823 | 0 | 81 | MALE | unknow | Stage IIB | T2 | M0 | N1 |
| TCGA-44-6146 | 728 | 0 | 64 | MALE | unknow | Stage IIB | T3 | M0 | N0 |
| TCGA-55-8513 | 791 | 0 | 77 | FEMALE | unknow | Stage IIB | T3 | MX | N0 |
| TCGA-MP-A5C7 | 2248 | 0 | 76 | FEMALE | unknow | Stage IB | T2 | M0 | N0 |
| TCGA-75-6214 | 1115 | 1 | unknow | FEMALE | unknow | Stage IIIA | T2 | M0 | N2 |
| TCGA-86-7713 | 1157 | 0 | 70 | MALE | unknow | Stage IIA | T2b | M0 | N0 |
| TCGA-75-6212 | 1516 | 1 | unknow | FEMALE | unknow | Stage IIB | T2 | M0 | N1 |
| TCGA-62-8394 | 139 | 1 | 65 | FEMALE | unknow | Stage IIIB | T4 | M0 | N2 |
| TCGA-64-5778 | 1305 | 0 | 60 | MALE | unknow | Stage IB | T2 | M0 | N0 |
| TCGA-97-8174 | 164 | 1 | 67 | MALE | unknow | Stage IIA | T2b | M0 | N0 |
| TCGA-50-8459 | 1119 | 0 | 68 | MALE | unknow | Stage IIB | T3 | M0 | N0 |
| TCGA-50-8457 | 1125 | 0 | 63 | FEMALE | unknow | Stage IA | T1a | M0 | N0 |
| TCGA-97-A4M1 | 601 | 0 | 52 | FEMALE | unknow | Stage IA | T1a | M0 | N0 |
| TCGA-78-7166 | 258 | 1 | 84 | MALE | unknow | Stage IIB | T2 | M0 | N1 |
| TCGA-83-5908 | 824 | 0 | 59 | FEMALE | unknow | Stage IA | T1 | M0 | N0 |
| TCGA-05-4426 | 791 | 0 | 71 | MALE | unknow | Stage IB | T2 | M0 | N0 |
| TCGA-55-7995 | 889 | 0 | 73 | FEMALE | unknow | Stage IA | T1b | M0 | N0 |
| TCGA-93-8067 | 186 | 0 | 77 | MALE | unknow | Stage IB | T2a | MX | N0 |
| TCGA-55-7284 | 243 | 1 | 74 | MALE | unknow | Stage IIB | T3 | MX | N0 |
| TCGA-55-1595 | 1479 | 0 | 74 | FEMALE | unknow | Stage IA | T1 | M0 | N0 |
| TCGA-44-2655 | 1324 | 0 | 65 | FEMALE | unknow | Stage IA | T1 | M0 | N0 |
| TCGA-69-8453 | 813 | 0 | 77 | MALE | unknow | Stage IIB | T3 | MX | N0 |
| TCGA-91-6835 | 79 | 0 | 81 | FEMALE | unknow | Stage IA | T1 | M0 | N0 |
| TCGA-95-8039 | 830 | 0 | 72 | MALE | unknow | Stage IA | T1 | MX | N0 |
| TCGA-97-7938 | 18 | 1 | 76 | FEMALE | unknow | Stage IA | T1a | MX | N0 |
| TCGA-69-7978 | 134 | 0 | 59 | MALE | unknow | Stage IIB | T2b | MX | N1 |
| TCGA-55-7994 | 603 | 0 | 81 | MALE | unknow | Stage IIB | T3 | MX | N0 |
| TCGA-55-8619 | 416 | 0 | 72 | FEMALE | unknow | Stage IIB | T3 | MX | N0 |
| TCGA-78-7149 | 3940 | 0 | 71 | MALE | unknow | Stage IIIB | T4 | M0 | N0 |
| TCGA-38-4631 | 354 | 1 | 72 | FEMALE | unknow | Stage IB | T2 | M0 | N0 |
| TCGA-44-2662 | 1280 | 0 | 65 | MALE | unknow | Stage IB | T2 | M0 | N0 |
| TCGA-MP-A4SV | 2620 | 1 | 67 | MALE | unknow | Stage IB | T2 | M0 | N0 |
| TCGA-L9-A7SV | 565 | 0 | 69 | MALE | unknow | Stage IIA | T2a | M0 | N1 |
| TCGA-93-A4JQ | 526 | 0 | 49 | MALE | unknow | Stage IA | T1b | MX | N0 |
| TCGA-35-3615 | 14 | 0 | 57 | MALE | unknow | Stage IB | T2 | M0 | N0 |
| TCGA-05-4418 | 274 | 1 | 69 | MALE | unknow | Stage IIIA | T3 | M0 | N2 |
| TCGA-05-4249 | 1523 | 0 | 67 | MALE | unknow | Stage IB | T2 | M0 | N0 |
| TCGA-MN-A4N4 | 1175 | 0 | 57 | MALE | unknow | Stage IA | T1b | M0 | N0 |
| TCGA-73-4658 | 1600 | 1 | 80 | FEMALE | unknow | Stage IB | T2 | M0 | N0 |
| TCGA-44-A4SS | 415 | 0 | 73 | MALE | unknow | Stage IA | T1b | M0 | N0 |
| TCGA-95-A4VP | 605 | 0 | 66 | FEMALE | unknow | Stage IIIA | T2b | M0 | N2 |
| TCGA-55-7914 | 187 | 1 | 71 | FEMALE | unknow | Stage IIA | T1b | MX | N1 |
| TCGA-78-7145 | 826 | 1 | 52 | FEMALE | unknow | Stage IV | T4 | M1 | N1 |
| TCGA-55-8092 | 154 | 1 | 75 | MALE | unknow | Stage IIB | T3 | MX | N0 |
| TCGA-MP-A4TE | 896 | 1 | 56 | MALE | unknow | Stage IIA | T2b | MX | N0 |
| TCGA-MP-A4T4 | 2617 | 1 | 68 | FEMALE | unknow | Stage IIB | T2 | M0 | N1 |
| TCGA-55-8203 | 547 | 0 | 69 | FEMALE | unknow | Stage IA | T1b | M0 | N0 |
| TCGA-55-6712 | 171 | 1 | 71 | MALE | unknow | Stage IIA | T2a | MX | N1 |
| TCGA-49-6743 | 1621 | 0 | 81 | FEMALE | unknow | Stage IIIA | T1 | MX | N2 |
| TCGA-73-4668 | 467 | 0 | 66 | FEMALE | unknow | Stage IIB | T2 | M0 | N1 |
| TCGA-67-3770 | 610 | 0 | 70 | FEMALE | unknow | Stage IA | T1 | M0 | N0 |
| TCGA-64-1680 | 1126 | 0 | 63 | MALE | unknow | Stage IV | T2a | M1 | N2 |
| TCGA-05-4398 | 1431 | 0 | 47 | FEMALE | unknow | Stage IIIB | T4 | M0 | N3 |
| TCGA-78-7155 | 1171 | 1 | 68 | MALE | unknow | Stage IB | T2 | M0 | N0 |
| TCGA-78-7152 | 1215 | 1 | 65 | MALE | unknow | Stage IB | T2 | M0 | N0 |
| TCGA-75-6211 |  | 1 | unknow | FEMALE | unknow | Stage IB | T2 | M0 | N0 |
| TCGA-MP-A4T6 | 1790 | 1 | 76 | FEMALE | unknow | Stage IIIA | T1 | MX | N2 |
| TCGA-55-A57B | 546 | 0 | 80 | FEMALE | unknow | Stage IA | T1b | M0 | N0 |
| TCGA-44-2656 | 1429 | 0 | 59 | MALE | unknow | Stage IB | T2 | M0 | N0 |
| TCGA-93-A4JO | 33 | 1 | 70 | MALE | unknow | Stage IA | T1a | MX | N0 |
| TCGA-78-7535 | 949 | 1 | 45 | MALE | unknow | Stage IB | T2 | M0 | N0 |
| TCGA-62-8398 | 444 | 1 | 55 | MALE | unknow | Stage IIIA | T2 | M0 | N2 |
| TCGA-91-6840 | 372 | 0 | 59 | FEMALE | unknow | Stage IA | T1b | M0 | N0 |
| TCGA-75-5122 |  | 1 | unknow | MALE | unknow | Stage IB | T2 | M0 | N0 |
| TCGA-99-8025 | 1060 | 0 | 72 | FEMALE | unknow | Stage IIIA | T3 | M0 | N2 |
| TCGA-55-8207 | 977 | 0 | 73 | MALE | unknow | Stage IB | T2a | MX | N0 |
| TCGA-55-6969 | 1239 | 0 | 52 | MALE | unknow | Stage IB | T2 | M0 | N0 |
| TCGA-64-5774 | 2676 | 0 | 60 | MALE | unknow | Stage IB | T2 | M0 | N0 |
| TCGA-O1-A52J | 1798 | 1 | 74 | FEMALE | unknow | Stage IA | T1 | MX | N0 |
| TCGA-86-6851 | 179 | 0 | 73 | FEMALE | unknow | Stage IIA | T1b | M0 | N1 |
| TCGA-62-8395 | 1216 | 0 | 80 | FEMALE | unknow | Stage IIB | T3 | M0 | N0 |
| TCGA-86-A4P7 | 415 | 0 | 63 | FEMALE | unknow | Stage IB | T2a | M0 | N0 |
| TCGA-91-6831 | 310 | 0 | 66 | MALE | unknow | Stage IB | T2 | MX | N0 |
| TCGA-05-4420 | 912 | 0 | 41 | MALE | unknow | Stage IB | T2 | M0 | N0 |
| TCGA-05-4395 | 0 | 1 | 76 | MALE | unknow | Stage IIIB | T4 | M0 | N2 |
| TCGA-67-6217 | 422 | 0 | 73 | FEMALE | unknow | Stage IIA | T2a | M0 | N1 |
| TCGA-44-4112 | 808 | 1 | 60 | FEMALE | unknow | Stage IB | T2a | M0 | N0 |
| TCGA-49-AAR2 | 2224 | 0 | 64 | MALE | unknow | Stage IB | T2 | MX | N0 |
| TCGA-62-A46O | 1454 | 1 | 65 | FEMALE | unknow | Stage IB | T2 | M0 | N0 |
| TCGA-44-7660 | 592 | 0 | 72 | MALE | unknow | Stage IB | T2 | MX | N0 |
| TCGA-44-3919 | 1026 | 1 | 71 | FEMALE | unknow | Stage IA | T1 | M0 | N0 |
| TCGA-44-6775 | 705 | 0 | 72 | FEMALE | unknow | Stage IB | T2a | MX | N0 |
| TCGA-97-7554 | 775 | 0 | 83 | FEMALE | unknow | Stage IIIA | T2a | M0 | N2 |
| TCGA-86-6562 | 376 | 1 | 52 | MALE | unknow | Stage IIA | T2a | M0 | N1 |
| TCGA-55-6642 | 2449 | 0 | 63 | MALE | unknow | Stage IB | T2 | MX | N0 |
| TCGA-62-A471 | 1246 | 0 | 64 | MALE | unknow | Stage IIB | T2b | M0 | N1 |
| TCGA-05-5715 | 62 | 0 | 69 | FEMALE | unknow | Stage IB | T2a | M0 | N0 |
