# Supplementary material for: Characteristics of Fatty Acid Metabolism in Lung Adenocarcinoma to Guide Clinical Treatment
Source: Front Immunol. 2022 Jul 1;13:916284. doi: 10.3389/fimmu.2022.916284 (PMC9289740; doi:10.3389/fimmu.2022.916284)
Supplement: Supplementary Table 2 — 309 fatty acid metabolism-related genes were obtained from previous studies and GSEA. [file Table_2.doc]

309 fatty acid metabolism-related genes

| FADS2 |
| --- |
| RAP1GDS1 |
| SDHA |
| ALDH2 |
| ACSBG1 |
| HSD17B4 |
| HACD1 |
| HSD17B3 |
| PTGES2 |
| DLD |
| CBR1 |
| ADIPOR2 |
| HPGD |
| CD1D |
| ERP29 |
| PTGES |
| AADAT |
| PDHA1 |
| ACAA2 |
| ACSF3 |
| MAOA |
| PRDX6 |
| CD36 |
| PRKAG2 |
| ACADVL |
| ELOVL2 |
| ALOX15B |
| PON1 |
| MDH1 |
| PECR |
| PPT2 |
| GPX1 |
| ENO3 |
| ACBD4 |
| CA6 |
| CEL |
| CYP4F8 |
| ACBD6 |
| MORC2 |
| SDHC |
| UBE2L6 |
| CYP2C9 |
| CYP4A22 |
| ADH1A |
| DPEP3 |
| ACBD5 |
| AWAT1 |
| ALOXE3 |
| SLC25A1 |
| EHHADH |
| ACACA |
| ALDH1A1 |
| ACAA1 |
| CA4 |
| OLAH |
| PRKAA2 |
| ACOT7 |
| ACOT8 |
| ABCD1 |
| ACSL3 |
| GLUL |
| ACOT9 |
| NCAPH2 |
| ADH5 |
| HACD2 |
| OSTC |
| CYP1B1 |
| CYP4F22 |
| FADS1 |
| HMGCS2 |
| IDH3G |
| ALOX15 |
| MECR |
| CPOX |
| HSD17B11 |
| ACSM6 |
| HACD4 |
| FAAH |
| CYP2U1 |
| NTHL1 |
| MIX23 |
| SDHD |
| HSPH1 |
| KMT5A |
| MID1IP1 |
| ALDH1B1 |
| PPARD |
| HTD2 |
| SUCLA2 |
| ADH4 |
| UROD |
| PTGDS |
| HADHA |
| ABCC1 |
| PTGR1 |
| DHCR24 |
| HCCS |
| FABP2 |
| GPD1 |
| PHYH |
| CYP2C8 |
| ECI1 |
| CYP2J2 |
| RXRA |
| THEM4 |
| FABP1 |
| PDHB |
| PTGR2 |
| ACOXL |
| NDUFAB1 |
| CBR3 |
| MCEE |
| PTGES3 |
| CRYZ |
| PCTP |
| THEM5 |
| CPT1B |
| LTA4H |
| CYP8B1 |
| ADH1C |
| HSD17B8 |
| BLVRA |
| SUCLG2 |
| THRSP |
| ACOX3 |
| ECH1 |
| CYP1A2 |
| GCDH |
| ELOVL3 |
| PON3 |
| TDO2 |
| ACADS |
| SCD5 |
| YWHAH |
| GGT5 |
| TBXAS1 |
| AMACR |
| PCCA |
| ACOT11 |
| PTGS1 |
| EPHX1 |
| ACSBG2 |
| ACSF2 |
| ALDH3A1 |
| ME1 |
| FASN |
| DPEP1 |
| GPX2 |
| GAD2 |
| AKR1C3 |
| CYP4F3 |
| MAPKAPK2 |
| CBR4 |
| MCAT |
| ODC1 |
| ALAD |
| CIDEA |
| ELOVL4 |
| CA2 |
| RDH11 |
| AOC3 |
| GGT1 |
| UROS |
| CYP2C19 |
| LDHA |
| ACADM |
| ACOT13 |
| HADHB |
| ADH6 |
| ACADSB |
| ACSL1 |
| ACAT1 |
| G0S2 |
| ELOVL1 |
| CROT |
| GABARAPL1 |
| ALOX5AP |
| NSDHL |
| FMO1 |
| ACAT2 |
| ALOX5 |
| PTS |
| TECRL |
| GPX4 |
| CPT1A |
| FAAH2 |
| ECHS1 |
| GSTZ1 |
| ACSS1 |
| HACD3 |
| IDH3B |
| GRHPR |
| FH |
| ETFDH |
| ACSM3 |
| PLA2G4A |
| ACAD10 |
| HADH |
| ACBD7 |
| SCP2 |
| AUH |
| D2HGDH |
| SLC22A5 |
| H2AZ1 |
| SMS |
| XIST |
| ALOX12B |
| ELOVL5 |
| NUDT7 |
| ALDOA |
| VNN1 |
| CYP4B1 |
| MMUT |
| ACO2 |
| UGDH |
| HSD17B12 |
| MDH2 |
| PPT1 |
| DPEP2 |
| EPHX2 |
| ACOT6 |
| PON2 |
| PSME1 |
| ACOT4 |
| PTGS2 |
| PTPRG |
| HAO2 |
| ACOT2 |
| SLC27A3 |
| SERINC1 |
| DLST |
| HSD17B10 |
| ACLY |
| TECR |
| HMGCS1 |
| ECI2 |
| BPHL |
| METAP1 |
| APEX1 |
| ELOVL6 |
| MIF |
| RETSAT |
| NUDT19 |
| HPGDS |
| HSDL2 |
| ADSL |
| PCCB |
| ACSL5 |
| SCD |
| RDH16 |
| PRXL2B |
| DECR2 |
| SLC25A20 |
| ACOX1 |
| ACSL6 |
| ACSL4 |
| AQP7 |
| IL4I1 |
| ACADL |
| PTGIS |
| ALDH3A2 |
| REEP6 |
| HMGCL |
| ALDH7A1 |
| ACAD11 |
| INMT |
| ACACB |
| CPT1C |
| CYP4F11 |
| ADH1B |
| ACOX2 |
| DECR1 |
| ENO2 |
| CYP4F2 |
| IDI1 |
| ALDH9A1 |
| HIBCH |
| LTC4S |
| ELOVL7 |
| ALOX12 |
| MMAA |
| NBN |
| LGALS1 |
| GAPDHS |
| MGLL |
| SLC27A2 |
| PPARA |
| HSP90AA1 |
| MLYCD |
| CYP1A1 |
| PCBD1 |
| SLC25A17 |
| BCKDHB |
| DBI |
| SUCLG1 |
| CPT2 |
| ACOT12 |
| HACL1 |
| ADH7 |
| GPD2 |
| TP53INP2 |
| IDH1 |
| S100A10 |
| CRAT |
| HSD17B7 |
| CYP4A11 |
| ACOT1 |
| PRKAB2 |
| BMPR1B |
