# Supplementary material for: Characteristics of Fatty Acid Metabolism in Lung Adenocarcinoma to Guide Clinical Treatment
Source: Front Immunol. 2022 Jul 1;13:916284. doi: 10.3389/fimmu.2022.916284 (PMC9289740; doi:10.3389/fimmu.2022.916284)
Supplement: Supplementary Table 3 — Non-zero regression coefficients calculated using the LASSO Cox regression analysis. [file Table_3.doc]

non-zero regression

| Gene | Coef |
| --- | --- |
| ALDH2 | -0.045176076 |
| HACD1 | 0.132795044 |
| ELOVL2 | 0.187661335 |
| ENO3 | -0.121527774 |
| CEL | -0.035373665 |
| CA4 | -0.005391128 |
| CYP2U1 | -0.186734455 |
| LDHA | 0.288845242 |
| ALOX5AP | -0.070833471 |
| SMS | 0.17467022 |
| ALDOA | 0.105989392 |
| CYP4B1 | -0.046914421 |
| DPEP2 | -0.057066042 |
| ELOVL6 | 0.010533136 |
